# H7N7 Avian Influenza Virus Mutation from Low to High Pathogenicity on a Layer Chicken Farm in the UK

**DOI:** 10.3390/v13020259

**Published:** 2021-02-08

**Authors:** Alexander M. P. Byrne, Scott M. Reid, Amanda H. Seekings, Alejandro Núñez, Ana B. Obeso Prieto, Susan Ridout, Caroline J. Warren, Anita Puranik, Vanessa Ceeraz, Stephen Essen, Marek J. Slomka, Jill Banks, Ian H. Brown, Sharon M. Brookes

**Affiliations:** 1 Department of Virology, Animal and Plant Health Agency-Weybridge, Woodham Lane, New Haw, Addlestone, Surrey KT15 3NB, UK; Alexander.Byrne@APHA.gov.uk (A.M.P.B.); Amanda.Seekings@APHA.gov.uk (A.H.S.); Caroline.Warren@APHA.gov.uk (C.J.W.); A.Puranik@VMD.gov.uk (A.P.); Vanessa.Ceeraz@APHA.gov.uk (V.C.); Steve.Essen@APHA.gov.uk (S.E.); Marek.Slomka@APHA.gov.uk (M.J.S.); Jillybanks@btinternet.com (J.B.); Ian.Brown@APHA.gov.uk (I.H.B.); Sharon.Brookes@APHA.gov.uk (S.M.B.); 2 Pathology and Animal Sciences, Animal and Plant Health Agency-Weybridge, Woodham Lane, New Haw, Addlestone, Surrey KT15 3NB, UK; Alejandro.Nunez@APHA.gov.uk; 3 Field Delivery, Animal and Plant Health Agency-Manchester, Manchester Airport, Manchester M90 5PZ, UK; Ana.Obeso@APHA.gov.uk; 4 Field Delivery, Animal and Plant Health Agency-Crewe, Hornbeam House, Electra Way, Crewe, Cheshire CW1 6GJ, UK; Sue.Ridout@APHA.gov.uk

**Keywords:** H7, avian influenza, outbreak, low pathogenicity, high pathogenicity, poultry

## Abstract

Avian influenza virus (AIV) subtypes H5 and H7 are capable of mutating from low to high pathogenicity strains, causing high mortality in poultry with significant economic losses globally. During 2015, two outbreaks of H7N7 low pathogenicity AIV (LPAIV) in Germany, and one each in the United Kingdom (UK) and The Netherlands occurred, as well as single outbreaks of H7N7 high pathogenicity AIV (HPAIV) in Germany and the UK. Both HPAIV outbreaks were linked to precursor H7N7 LPAIV outbreaks on the same or adjacent premises. Herein, we describe the clinical, epidemiological, and virological investigations for the H7N7 UK HPAIV outbreak on a farm with layer chickens in mixed free-range and caged units. H7N7 HPAIV was identified and isolated from clinical samples, as well as H7N7 LPAIV, which could not be isolated. Using serological and molecular evidence, we postulate how the viruses spread throughout the premises, indicating potential points of incursion and possible locations for the mutation event. Serological and mortality data suggested that the LPAIV infection preceded the HPAIV infection and afforded some clinical protection against the HPAIV. These results document the identification of a LPAIV to HPAIV mutation in nature, providing insights into factors that drive its manifestation during outbreaks.

## 1. Introduction

Avian Influenza (AI) caused by subtypes H5 and H7 is a notifiable avian disease (NAD) and a severe threat to the poultry industry globally, whilst the emergence of novel avian influenza virus (AIV) strains from wild bird reservoirs is a constant threat to animal and human health. From 1959 to 2017, the United Kingdom (UK) had 11 outbreaks of low pathogenicity AIV (LPAIV, subtypes H5 and H7) and 13 outbreaks of high pathogenicity AIV (HPAIV) [1,2,3,4]. Notably, these have included two LPAIV to HPAIV mutational events of the H7N7 subtype in layer chickens, occurring in 2008 [4] and 2015 [5]. During both events only the HPAIV was isolated from the premises; however, viral RNA containing hemagglutinin (HA) cleavage site (CS) motifs consistent with a LPAIV were found. Acquisition of HA CS alterations in LPAIV to become HPAIV occurs in part due to the insertion of basic amino acids [1]. These insertions make the HA proteolytic CS more accessible to ubiquitous intracellular proteases, allowing for systemic spread of the virus within the host [1]. Nevertheless, observing this mutation on a single premises during an outbreak is rare and three other UK H7 LPAIVs (H7N3 in 2006 [6], H7N2 in 2007 [7], and H7N7 in 2015 [3]) did not mutate to HPAIVs.

During 2015, there were four poultry outbreaks of H7N7 LPAIV in Europe, one in the UK [3], one in The Netherlands, and two in Germany [8]. There were also two outbreaks of H7N7 HPAIV, one in the UK [5] and one in Germany [8], both linked to earlier LPAIV incursions. The H7N7 HPAIV outbreak in Germany emerged from a H7N7 LPAIV precursor that had caused an outbreak in a farm located only 400 m away [8], whereas in the UK, this mutation occurred on the same farm. For both HPAIV outbreaks, the onward spread was limited but such identifications of LPAIV to HPAIV mutations highlight the importance of early detection and rapid implementation of control measures to limit within-farm, as well as onward spread of disease to other premises. These mutation events may also be the product of the selective pressures induced by rapid and efficient within-flock transmission, in combination with host switching due to the recent introduction from waterfowl to gallinaceous poultry [8,9].

In this report, we describe the clinical, epidemiological, and virological investigations during the 2015 UK H7N7 HPAIV outbreak in layer chickens on a farm with mixed free-range and caged units. This investigation provides further evidence for the LPAIV to HPAIV mutation occurring at a single poultry premises, following the previously-reported LPAIV to HPAIV mutation event in 2008 [4], and ultimately leading to a better understanding as to how and why some H7 LPAIVs appear to readily mutate to HPAIV while others do not.

## 2. Materials and Methods

### 2.1. Epidemiological Investigation and Sample Collection

Following a notification of suspicion of a NAD by a local private veterinary surgeon (PVS), epidemiological investigations were carried out on a farm consisting of ten sheds housing layer chickens at 67 weeks of age in the county of Lancashire in the northwest of England, according to the European Council Directive 2005/94/EC [10]. These investigations were aimed at determining the potential sources of infection and spread to other premises. This involved an on-farm assessment and detailed interviews with the farm manager/owner and the PVS. Additional information was obtained from production and staff records, invoices, and movement data for transport vehicles.

Samples for disease investigation were collected from the infected premises (IP) in three separate sample sets (Table 1). Sample Set 1 consisted of 11 carcasses from two sheds that were submitted by the PVS to the Animal and Plant Health Agency (APHA) for post-mortem examination (PME) as part of a preliminary investigation due to increased mortality. Sample Set 2 was collected as part of the follow-up NAD investigation and consisted of oropharyngeal (OP) swabs, cloacal (C) swabs, and clotted bloods (20 of each) taken from birds in five separate sheds and was used to confirm the presence of NAD on the premises. Sample Set 3 was collected after NAD confirmation, during statutory culling and included OP and C swabs, clotted bloods (60 of each) and two carcasses, each taken from nine sheds. The third sample set was collected to enable further understanding of the underlying epidemiology of the outbreak.

### 2.2. Pathology and Immunohistochemistry (IHC)

Tissue samples were fixed in 10% (*v*/*v*) buffered formalin for a minimum period of five days and routinely processed for histopathology. Influenza A viral antigen was detected by immunohistochemistry (IHC) as described previously [2].

### 2.3. RNA Extraction and Real-Time Reverse Transcription-PCR (RRT-PCR)

RNA was extracted from swab fluids and tissue homogenates; brain, lung and trachea, mixed viscera (heart, liver, kidney, and spleen) and intestines, as described previously [11].

Extracted RNA was tested using AIV real-time reverse-transcription PCR (RRT-PCR) assays for generic influenza A virus detection targeting the matrix (M) gene [4,12], and for specific detection of H5 [13] or H7 [11] AIVs. RNA was also tested using one avian orthoavulavirus type 1 (AOAV-1) RRT–PCR assay [14]. Samples were considered to be RRT-PCR-positive by the AIV RRT-PCRs if the cycle threshold (Ct) value was less than or equal to 36.0, whilst samples were positive by the AOAV-1 RRT-PCR assay if the Ct value was less than or equal to 37.0. An individual bird was considered positive if either the OP or C swab was determined to be positive by RRT-PCR. For analyses comparing the route of viral shedding and PCR efficacy, all Ct values were included even if greater than these validated thresholds. Any samples for which the RRT-PCR result was “No Ct”, was interpreted as a Ct value of 40.0 for these analyses.

### 2.4. Sequencing and Phylogenetic Analysis

For molecular pathotyping, conventional reverse-transcription (RT)-PCR and Sanger sequencing was carried out across the HA CS using the Gk7.3 and Gk7.4 primer pair as described previously [11,15]. Nucleotide sequence analysis and alignment was carried out using the Lasergene software suite version 10 (DNASTAR, Madison, USA).

For whole-genome sequencing (WGS), viral RNA was extracted manually [11] but without the addition of carrier RNA. Double-stranded cDNA was generated using the cDNA Synthesis System (Roche, Burgess Hill, UK) according to the manufacturer’s instructions. This was quantified using the QuantiFLUOR dsDNA system (Promega, Southampton, UK) and 1 ng used as the template for preparation of the sequencing library using the NexteraXT kit (Illumina, Cambridge, UK). Sequencing libraries were run on a MiSeq sequencer (Illumina, Cambridge, UK) with 2 × 150 base paired-end reads. The raw sequence reads were assembled using a custom script, FluSeqID (https://github.com/ellisrichardj/FluSeqID (accessed on 1 June 2020)).

Related AIV genomic sequences of contemporary European H7N7 viruses and those available in public databases were identified using BLAST to search for common ancestors from the EPIFlu database on the GISAID website (https://platform.gisaid.org/epi2/ (accessed on 1 June 2020)). Gene sequences were aligned with MAFFT version 7.427 [16]. Phylogenetic trees were inferred using the maximum-likelihood approach in IQ-Tree version 1.6.10 [17] with ModelFinder [18] to infer the appropriate phylogenetic model and 1000 bootstrap replicates [19]. Phylogenetic trees were visualized, edited, and annotated using MEGA7 [20]. Pairwise sequence identity was determined using the Sequence Identity and Similarity (SIAS) tool (http://imed.med.ucm.es/Tools/sias.html (accessed on 1 June 2020)).

### 2.5. Virus Isolation

Virus isolation was performed using 9 to 11 day-old specified pathogen free (SPF) embryonated fowls’ eggs for pools of five OP or C swab groups according to internationally-recognized European Union (EU) and OIE methods [21,22].

### 2.6. Hemagglutination Inhibition (HI) and Neuraminidase Inhibition (NI) Tests

After decanting from clotted bloods, sera were screened by hemagglutination inhibition (HI) tests to detect virus subtype-specific antibodies against H5 or H7 AIV and AOAV-1 antigens [22,23]. The following H5 and H7 AIV antigens described for use in the annual AI poultry serosurveillance programme in all EU Member States were used: A/teal/England/73942805/06 (H5N3); primary H5 antigen, A/turkey/England/647/77 (H7N7); primary H7 antigen and A/African starling/England/983/79 (H7N1); secondary H7 antigen [23]. Serum samples with a reciprocal HI titer of greater than or equal to 16 were considered positive [22]. Virus subtyping by the HI and neuraminidase inhibition (NI) tests were performed on an egg-amplified isolate using a panel of typing antisera according to standard methods [22].

### 2.7. Intravenous Pathogenicity Index (IVPI)

An intravenous pathogenicity index (IVPI) test was performed according to the standard method [22] to determine the pathogenicity of the index AIV isolate from the IP.

### 2.8. Statistical Analyses

All statistical analyses were performed using Prism version 7 (GraphPad, San Diego, CA, USA).

## 3. Results

### 3.1. Case Description

**Figure 1:**
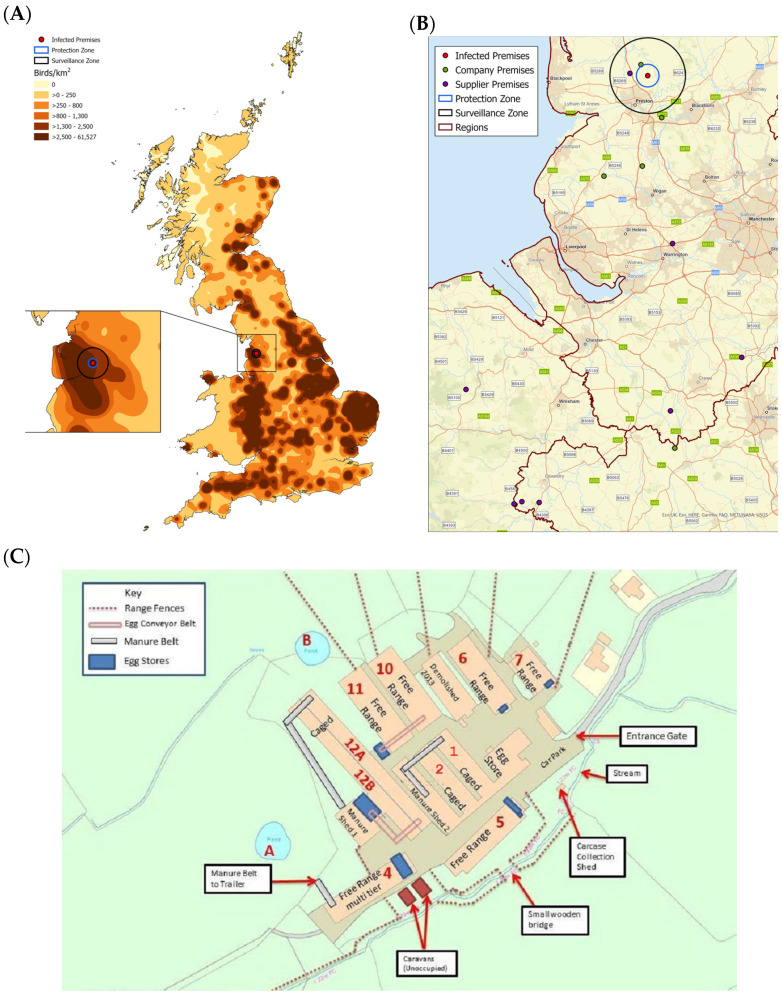
The IP was located in a poultry-dense area in the northwest of England. (**A**) A map showing the density of poultry across Great Britain. The map was created using an extract of the Animal and Plant Health Agency (APHA) animal holding database at the time of the investigation. The density of birds was determined using the kernel density function in ArcGIS using a 15 km search radius and output cell size of 1 km. The location of the IP, 3 km protection zone (PZ) and 10 km surveillance zone (SZ) are also shown. (**B**) A map showing the locations of linked premises, represented by green, company-owned premises, and purple, supplier premises, dots. The location of the IP, 3 km PZ and 10 km SZ and the regions are also shown. (**C**) A diagram depicting the site plan of the IP. The sheds are labelled accordingly with their numbers and the type of housing. The location of range fences, egg conveyor belts, manure belts and eggs stores, as well as ponds are also shown.

The IP was a family-run business located in a poultry-dense area in the county of Lancashire in the northwest of England (Figure 1A,B), containing 170,000 laying hens housed in ten sheds. Four sheds, numbered 1, 2, 12A, and 12B, housed 120,000 birds in enriched cages, with the remaining six sheds, numbered 4, 5, 6, 7, 10, and 11 housing a total of 50,000 free-range birds. The latter birds had access to ranges associated with each shed during the daytime (Figure 1C). None of the ranges were fenced-off as individual units, but mixing of poultry from different sheds was considered unlikely [5]. However, the ranges were uncovered and therefore freely accessible to wild birds. There were also two ponds located on the premises within the ranges of Sheds 4 and 11, which were frequented by ducks and other wildfowl.

An “all-in/all-out” production policy was operated on the premises, with all birds at 67 weeks-of-age. The flock arrived on site in July 2014 at 16 weeks-of-age, with no movement of live birds onto or off the site prior to July 2015. Seven linked premises owned by the same business included two pullet farms that supplied five commercial laying premises (Figure 1B). Two of the laying premises had co-located egg packing stations that received table eggs from six additional commercial free-range laying premises located in the same area for repackaging, distribution, and sale within the UK, but were not owned by the same business (Figure 1B). Whilst none of the linked premises were located within the 3 km protection zone (PZ) and only a single company-owned premises was located within the 10 km surveillance zone (SZ), there were 87 other premises located within the PZ, and 127 within the SZ.

### 3.2. Clinical Background and Submission of Samples

In caged Sheds 1 and 12A, there was a drop in egg production noted on 3 July 2015, coinciding with hot weather. This was followed by an increase in mortality during 4–5 July as temperatures decreased, and a sudden escalation in mortality on 6 July, with a total of 1800 birds dead across Sheds 1, 2, 10, 11, and 12A. The farm manager highlighted the increased mortality and contacted the PVS. Restrictions on the movement of eggs and vehicles were placed on the premises by the company, biosecurity was strengthened, and no eggs left the farm after 6 July.

On 7 July, the PVS visited the farm and began antibiotic treatment using tetracycline. Mortality at this point had reached approximately 2500 birds in total and a PME conducted by the PVS identified petechial hemorrhages in the spleen and liver. Sample Set 1, consisting of eleven carcasses were submitted by the PVS from Sheds 10 (free-range) and 12A (caged) to APHA on 7 July as part of a preliminary investigation (Table 1).

By the 8 July, over 3000 birds had died. Chickens in two free-range sheds (Sheds 10 and 11) and three caged sheds (Sheds 1, 2, and 12A) exhibited clinical signs, including diarrhea, hunching (dropped heads and eyes closed) and/or cyanosis around the wattles and combs. The PVS suspected NAD and an official veterinary investigation was instigated with restrictions served on the premises.

Sample Set 2 was collected from five sheds on 9 July as part of a follow-up investigation (Table 1). On 10 July, results from Sample Set 1 confirmed the presence of H7 AIV, which, in combination with the deteriorating clinical picture on the premises resulted in the UK Chief Veterinary Officer authorizing the culling of the birds on suspicion of NAD and establishment of a 10 km temporary control zone (TCZ). Culling commenced on 11 July, with Sample Set 3 being collected between 11–13 July from randomly-selected birds housed in nine sheds (Table 1) to allow further research into the epidemiological and virological factors underlying the outbreak. H7N7 HPAIV was confirmed on 13 July based on HA CS sequencing of samples in Sample Set 2, resulting in the replacement of the 10 km TCZ with a 3 km PZ and 10 km SZ (Figure 1B). Culling was completed on 14 July, with carcass disposal and preliminary cleaning and disinfection completed on 16 July. The PZ and SZ were merged on 7 August, and finally lifted on 16 August in the absence of further cases.

### 3.3. Pathology

**Figure 2:**
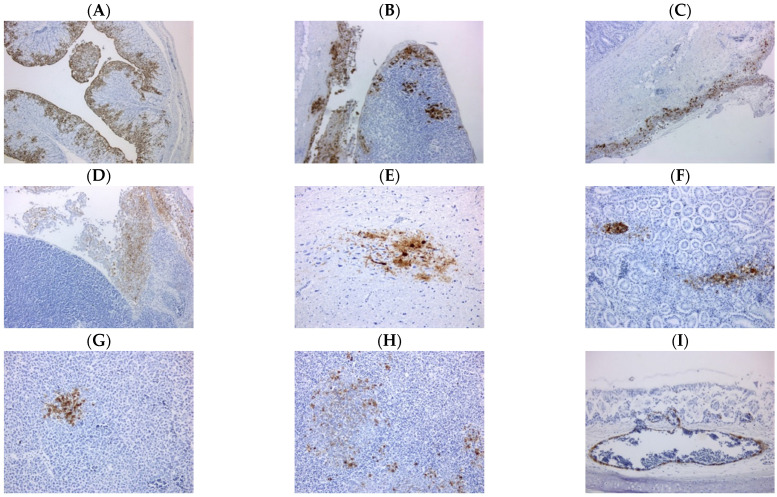
Immunohistochemistry (IHC) revealed systemic dissemination of influenza A viral antigen. Representative IHC images of tissue sections labelled against influenza A viral antigen (brown) taken from (**A**) oviduct, (**B**) pancreas and peritoneum, (**C**) jejunal peritoneum, (**D**) pancreatic surface, (**E**) brain, (**F**) kidney, (**G**) liver, (**H**) spleen and (**I**) tracheal vascular endothelium. The following magnifications were used: images (**A**,**B**) 50×, images (**C**,**D**) 100× and images (**E**–**I**) 200×.

Carcasses collected in Sample Sets 1 and 3 were examined by PME. Carcasses from three sheds were found to have significant gross pathology lesions including splenomegaly, multifocal splenic necrosis, and hemorrhagic ovarian follicles, with carcasses from the remaining sheds not showing specific changes. (Table S1). Histopathological presentation was diverse depending on the shed or individual bird, and included primarily fibrin necrotizing multifocal splenitis, necrotizing air sacculitis and chronic active coelomitis, and less frequently random multifocal necrotizing hepatitis and necrotizing pancreatitis, with intralesional demonstration of viral antigen (Table S1). IHC showed that carcasses from six sheds had disseminated viral antigen. Immunolabelled cells included: lymphoid tissue in the spleen and intestine, respiratory epithelium in trachea, lung and air sacs, pancreatic acinary cells, serosal membranes, oviduct epithelial cells, tubular epithelial cell in the kidney, neurons, glia and ependymal cells in the brain, and occasional endothelial cells and rare enterocytes in some birds (Figure 2). Viral antigen was only detected in the peritoneum and air sac of carcasses from Shed 5, whilst carcasses from Sheds 1 and 4 were negative by IHC (Table S1).

### 3.4. RRT-PCR Investigations

All standard tissue pools (brain, lung and trachea, intestines, and mixed viscera) collected from the carcasses in Sample Set 1 were positive for influenza A virus RNA, but negative for AOAV-1 (Table S2). All samples were RRT-PCR-negative for AIV H5 RNA, but positive for AIV H7 RNA.

This was corroborated by Sample Set 2 where all samples were negative for AOAV-1, but positive for influenza A and H7 RNA by RRT-PCR (Table 2). For this sample set, 50–70% of birds in each shed were RRT-PCR-positive for influenza A virus RNA, and 50–90% of samples were positive for H7 RNA.

With Sample Set 3, only Shed 4 was negative for H7 RNA by RRT-PCR, but the remaining sheds ranged from 2 to 90% positive at the whole bird level (Table 3). Carcasses taken prior to cull were also tested for H7 by RRT-PCR, with carcasses from five sheds showing systemic viral distribution (Table S3). As the RRT-PCR results from Sample Set 2 were AOAV-1- and H5-negative, Sample Set 3 was only tested by the H7 RRT-PCR assay.

**Figure 3:**
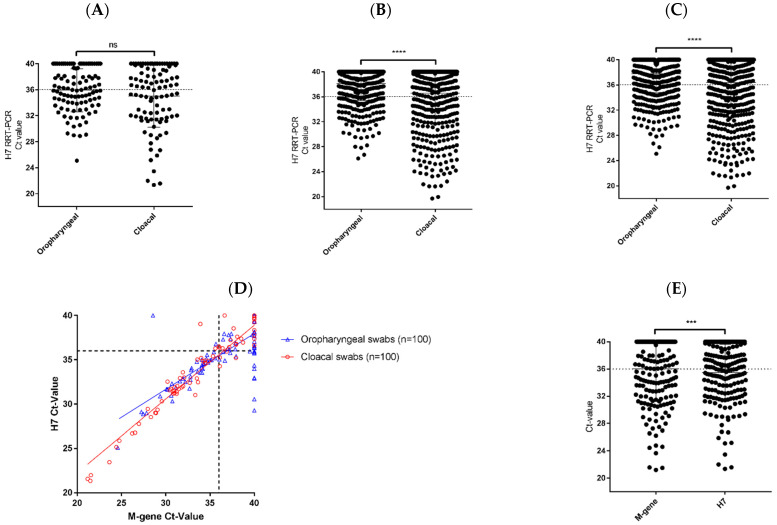
H7N7 high pathogenicity avian influenza virus (HPAIV) is shed predominantly via the cloacal route. Oropharyngeal (OP) and cloacal (C) swabs were tested by H7 RRT-PCR to determine the level of viral RNA present in the samples, as represented by the Ct value. The OP and C swab H7 RNA levels from (**A**) Sample Set 2 (N = 100, per swab type), (**B**) Sample Set 3 (N = 540, per swab type) and (**C**) all positive swab samples from Sample Sets 2 and 3 (N = 640, per swab type) were compared. (**D**) OP and C swabs in Sample Set 2 (N = 100, per swab type) were tested by M-gene and H7 RRT-PCRs and the correlation of the Ct values between assays was assessed using linear regression analysis or (**E**) direct comparison (N = 200, per assay). The Ct values were compared between OP and C swabs, or different RRT-PCR assays, using a Paired T-test for all samples. ns, not significant *p* > 0.05, *** *p* < 0.001 and **** *p* < 0.0001.

For Sample Set 2, the levels of H7 RNA detected in the OP and C swabs showed no significant differences (*p* = 0.0853) (Figure 3A), with no route of viral shedding being favored. However, from the swab samples collected in Sample Set 3, the amount of H7 RNA detected in the C swabs was significantly greater than that from the OP swabs (*p* < 0.0001) (Figure 3B). Overall, of the 1280 swab samples tested in Sample Sets 2 and 3, 383 (29.9%) were positive by H7 RRT-PCR. Comparison of all OP and C swabs showed that the amount of viral RNA was higher in the C swabs (Figure 3C); indicating that the virus was preferentially shed via the gastrointestinal route rather than the respiratory tract. The same was observed for two previous UK H7N7 outbreaks, one of H7N7 HPAIV in 2008 [4] and one of H7N7 LPAIV in 2015 [3] (Figure S1), despite these outbreaks being distinctly different in terms of pathogenicity. Comparison of the Ct values obtained by the M-gene and H7 RRT-PCR assays performed on the OP and C swabs from Sample Set 2 showed good qualitative correlation between the tests, especially around the diagnostic cut-off (Ct 36.0) (Figure 3D). However, Ct values obtained for the H7 RRT-PCR assay for this sample set were significantly lower than the Ct values obtained by the M-gene RRT-PCR assay, for all OP and C swab samples (*p* = 0.0004) (Figure 3E), suggesting that the H7 RRT-PCR assay was more sensitive.

### 3.5. Sequencing of the HA CS Motif

Sequencing of the HA CS of cecal tonsil samples from birds in Sheds 10 and 12A in Sample Set 1, revealed a HPAIV CS motif (Table S2). However, the samples from Shed 10 had the CS motif PEIPRHRK**G**RGLF (HP_G_), whereas the samples from Shed 12A had a CS motif PEIPRHRK**R**RGLF (HP_R_). HA CS sequencing results from Sample Sets 2 and 3 showed that both the HP_G_ and/or HP_R_ motifs were present in all sheds except Shed 4, where only a H7N7 LPAIV (PEIPKGRGLF) CS motif was detected (Table 2 and Table 3). Across Sample Sets 1, 2, and 3, the HP_G_ CS motif was identified in seven sheds, whereas the HP_R_ CS motif was detected in eight sheds (Table 4). The H7N7 LPAIV HA CS was only identified in three sheds, and interestingly in two of these sheds, Sheds 1 and 2, both H7N7 HPAIV and LPAIV HA CS motifs were detected.

### 3.6. Virus Isolation, Conventional Typing and IVPI

Hemagglutinating agents were isolated from at least one positive swab pool from each shed in Sample Set 2; however, it was not possible to isolate the H7N7 LPAIV identified in Sample Set 3. Of the viral isolates obtained, a single virus was selected as the prototype isolate for each shed (Table S4). The egg-amplified virus isolate obtained from the pooled C swabs from Shed 2, A/chicken/England/26352/2015, served as the index isolate for the outbreak and conventional subtyping identified the subtype as H7N7. The IVPI value obtained from this virus was 2.52, signifying that the AIV isolated was of high pathogenicity and confirming the molecular pathotype (Table 2, Table 3 and Table S2).

### 3.7. WGS and Phylogenetic Analysis

The full genome sequence of the index virus, A/chicken/England/26352/2015, was obtained using WGS (GISAID accession numbers: EPI62937-EPI623944) and was used for phylogenetic analyses.

**Figure 4:**
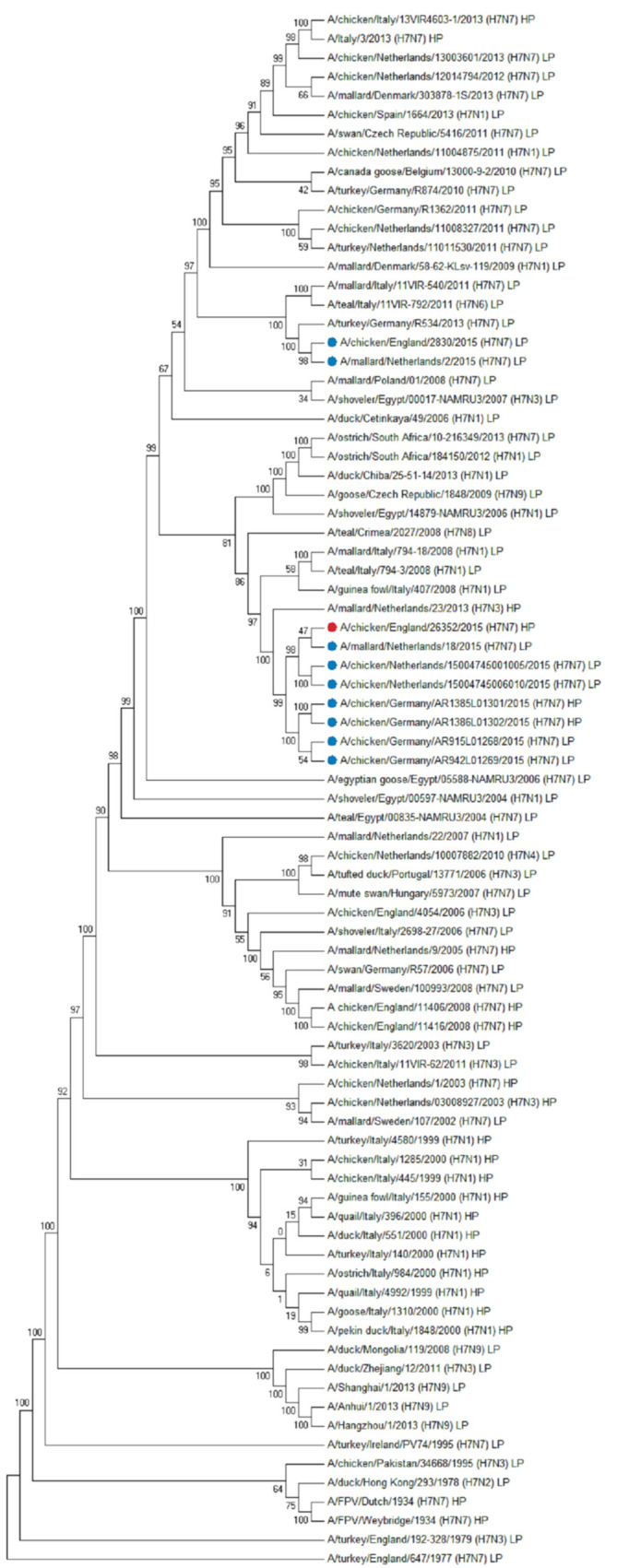
A/chicken/England/26352/2015 is related to contemporary European H7N7 viruses. Sequence comparisons of the HA gene of A/chicken/England/26352/2015 (H7N7) HPAIV identified A/mallard/Netherlands/18/2015 (H7N7) low pathogenicity AIV (LPAIV) to be the closest match at 99.16% identity. A/chicken/England/26352/2015 (H7N7) is indicated by a red circle, whilst European H7N7 influenza viruses from 2015 are indicated by blue circles. Phylogenetic analysis was conducted using a maximum-likelihood approach based on the GTR+F+G4 model. LP; low pathogenicity and HP; high pathogenicity.

The HA gene of A/chicken/England/26352/2015 was closely related to other contemporary European H7 AIVs, and genetically distinct from the H7N7 LPAIV that was isolated from UK poultry earlier in the same year [3] (Figure 4). Nucleotide sequence comparisons of the HA gene identified a H7N7 LPAIV isolated in The Netherlands in 2015, A/mallard/Netherlands/18/2015 as the closest related sequence at 99.16% identity. Comparisons of the nucleotide sequences of the other gene segments revealed phylogenetic relationships to contemporary H7N7 or other wild bird AIVs from Europe (Figure S2A–G).

Analysis of the A/chicken/England/26352/2015 full genome sequence for molecular determinants that may confer enhanced transmissibility or severe disease in mammals as defined by the Centre for Disease Control [24] revealed several polymorphisms (Table S5). These included polymorphisms involved in enhanced viral replication, increased binding of the HA protein to human receptors, and reduced susceptibility to oseltamivir and peramivir. However, mutations that confer reduced susceptibility to zanamivir or amantadine were not identified. Aside from these polymorphisms, there was no indication that this virus would have an increased tropism towards mammals.

### 3.8. Serological Investigation

**Figure 5:**
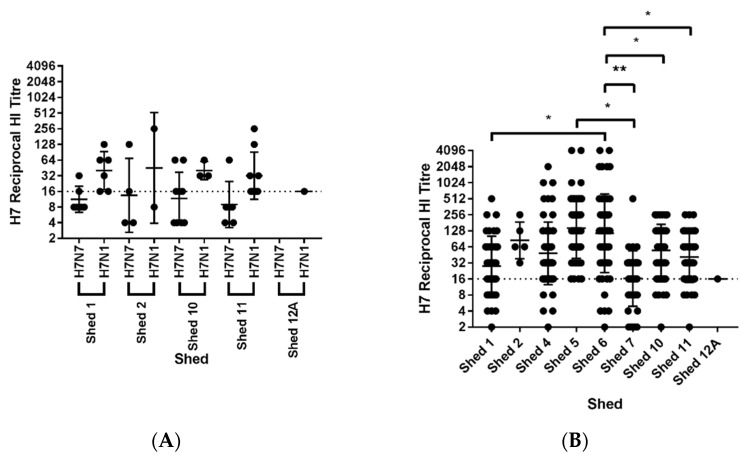
Antibodies towards H7 AIV were found in chickens from the IP. Sera in (**A**) Sample Set 2 and (**B**) Sample Set 3 collected from chickens at the IP were tested by HI assay. The total number of sera tested from each shed is provided in Table 5 (Sample Set 2) and Table 6 (Samples Set 3). The sera in Sample Set 2 were tested against the H7N7 and H7N1 primary and secondary AIV antigens, whereas sera from Sample Set 3 were tested against the H7N1 AIV antigen only. Graphs show geometric mean ± geometric SD. The reciprocal HI titers were compared between antigens and sheds using a One-Way ANOVA for all samples. * *p* < 0.05, ** *p* < 0.01.

In Sample Set 2, sera collected from all sheds were positive for antibodies to the H7 AIV antigens but negative for antibodies towards H5 AIV and AOAV-1 by HI assay (Table 5). The percentage of positive birds ranged from 7 to 45% within each shed and overall 28.6% of sera tested (N = 84) were positive for antibodies to the H7 AIV antigens. Selected birds showed reciprocal HI titers of up to 128 to the H7N7 AIV antigen and 256 to the H7N1 AIV antigen (Figure 5A).

When comparing the total number of sera with reciprocal HI titers of two or greater, there were no significant differences in the geometric mean of reciprocal HI titers between sheds. There was also no significant differences in the geometric mean of reciprocal HI titers of the sera to the H7N7 and H7N1 AIV antigens within sheds. However, when comparing the results across Sample Set 2, the geometric mean of reciprocal HI titers of sera were higher to the H7N1 AIV antigen (geometric mean 35.33), than to the H7N7 AIV antigen (geometric mean 11.16, *p* = 0.0002) (Figure S3A). For this reason, the sera collected in Sample Set 3 were only tested against the H7N1 AIV antigen, however, these findings reaffirm the importance of using both primary and secondary diagnostic antigens.

For sera from Sample Set 3, the percentage of positive birds ranged from 2 to 100% within each shed (Table 6), and overall 58.6% of the sera tested (N = 531) were positive for antibodies to the H7N1 AIV antigen, with selected birds showing reciprocal HI titers up to 4096 (Figure 5B). For all reciprocal HI titers of two or greater from Sample Set 3, Shed 6 showed a significantly higher geometric mean compared to Sheds 1, 7, 10, and 11 (*p* = 0.0112, *p* = 0.048, *p* = 0.0111, and *p* = 0.0129, respectively), the reciprocal HI titers from Shed 5 were also significantly greater than in Shed 7 (*p* = 0.0366) (Figure 5B).

When comparing the geometric mean of reciprocal HI titers towards the H7N1 AIV antigen, there was no significant difference between Sample Set 2 (geometric mean 35.33) and Sample Set 3 (geometric mean 56.08) (Figure S3B). The high proportion of seropositive birds in both sample sets supports prior infection with a H7 LPAIV and suggests infection before the emergence of the H7 HPAIV, as infection with H7 HPAIV in AI-naïve chickens typically causes rapid mortality without opportunity to mount a detectable humoral response [8].

To fully understand the infection status of the birds sampled on the premises, four broad categories were defined based on the RRT-PCR (Table 2 and Table 3) and serological (Table 5 and Table 6) data from Sample Sets 2 and 3 (Table 7). Category I: seronegative to H7 AIV antigens, consistent with no prior exposure to virus, and negative for H7 by RRT-PCR, suggesting no active infection. Birds in Category I had minimal or no exposure to AIV infection. Category II: seronegative to H7 AIV antigens, consistent with no prior exposure to virus, and H7 positive by RRT-PCR, therefore actively infected prior to seroconversion. Birds in Category II were in the early acute stage of AIV infection at the time of sampling. Category III: seropositive to H7 AIV antigens, consistent with exposure to virus at least seven to ten days prior to sampling, and H7 positive by RRT-PCR indicating that these birds were actively infected at the time of sampling. Birds in Category III may have been infected with the H7 LPAIV previously and were infected with H7 HPAIV at the time of sampling. Category IV: seropositive to H7 AIV antigens, consistent with exposure to virus at least seven to ten days prior to sampling and H7 negative by RRT-PCR, therefore not actively infected. Birds in Category IV would have cleared any previous AIV infection and were in the recovery phase at the time of sampling. Category I chickens were the majority in Sheds 7 and 12A, suggesting that more than 50% of the birds in these sheds experienced no infection with either the H7 LPAIV or HPAIV. However, these sheds were not completely free of infection with 40.7% of birds in Shed 7, and 47.5% of birds in Shed 12A demonstrating evidence of prior or active infection. Category II chickens were the majority in Shed 2, suggesting these animals were not infected with the H7 LPAIV and were actively infected with H7N7 HPAIV at the time of sampling. Category III chickens were highest in Shed 11, suggesting these birds were previously infected by the H7 LPAIV and were actively infected at the time of sampling. Finally, Category IV chickens were highest in Sheds 1, 4, 5, 6, and 10, suggesting that these birds had previously been infected with the H7 LPAIV and were not actively infected with H7N7 HPAIV at the time of sampling. However, in Sheds 1 and 10, the majority of animals were H7 RRT-PCR positive and therefore actively infected at the time of sampling. This indicates that in these sheds there was a combination of animals that had previously been infected with the H7 LPAIV and not the H7N7 HPAIV, as well as those who had been infected with the H7 LPAIV and were infected with the H7N7 HPAIV at the time of sampling.

## 4. Discussion

**Figure 6:**
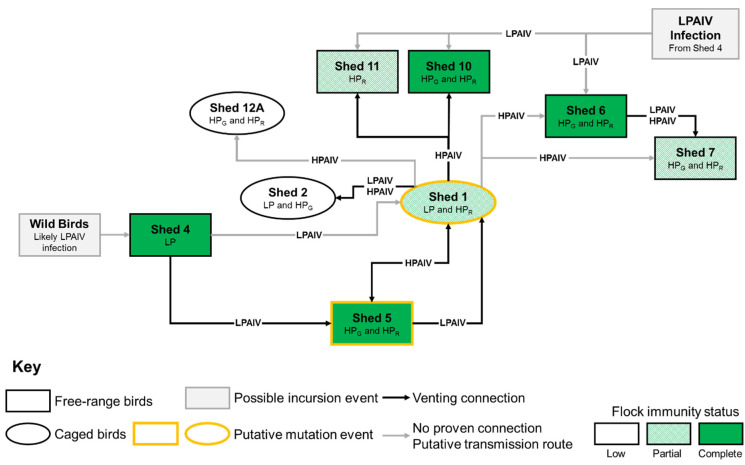
Putative transmission of H7N7 LPAIV and HPAIV through the IP. Molecular and serological investigations of samples collected in Sample Sets 2 and 3, were used to determine the immune and infection status of birds in different sheds on the IP to plot the putative spread of the virues through the different sheds. Flock immunity status per distinct epidemiological group was defined as follows: Low, virus detected and less than 40% seropositive animals; Partial, virus detected and 40–60% seropositive animals; Complete, some virus detected and greater than 60% seropositive animals. Shed 10 had 33% seropositives in Sample Set 2, but 82% in Sample Set 3 and was therefore considered to have complete flock immunity.

Evidence based on the clinical picture, laboratory results, epidemiological investigations and expert advice were considered in order to estimate the time of incursion and subsequent spread of virus during this H7N7 HPAIV outbreak in the UK. However, this estimation was complicated by the presence of the H7N7 LPAIV on the premises. Through the analysis of samples collected at cull (Sample Set 3, 11–13 July), it was possible to plot a time course of events by shed number on the IP, taking into account the different modalities of spread in free-range and caged birds (Figure 6).

Epidemiological tracing suggested that the most likely source of the outbreak was by direct or indirect contact with wild birds, particularly through wildfowl observed at the premises on the small ponds close to the ranges of free-range runs (Figure 1C). It was found that there was high potential for fomite transfer of virus on the premises and direct wild bird contact with the free-range birds. Production data and serological results suggest that this contact led to an incursion of a progenitor H7 LPAIV into Sheds 4 or 6 between 29 May and 19 June, 24–45 days prior to disease confirmation. Both Sheds 4 and 6 had high numbers of seropositive birds with high reciprocal HI titers, peaking at 2048 and 4096, respectively, and very few birds actively shedding virus, indicative of prior LPAIV infection which may have protected clinically against the HPAIV. However, Shed 4 was the most likely point of incursion based on the location of the shed in the vicinity of a pond frequented by wild waterfowl, with spread to Shed 6 occurring rapidly.

The LPAIV is likely to have then spread to Shed 7 shortly after Sheds 4 and 6, indicated by the significant proportion of birds in this shed demonstrating seroconversion, with a peak HI titer of 512, and no active viral shedding. This introduction was potentially mediated by human fomite transfer or by a vent connection with Shed 6 (Figure 6). The identification of the HP_G_ and HP_R_ CS motifs in Sheds 6 and 7, along with the lack of epidemiological evidence for multiple introductions of the LPAIV from wild birds or multiple mutation events, indicates that there was intra-farm spread and evolution of the HPAIV after mutation from LPAIV. However, the spread of the HPAIV within these sheds was limited, with very few birds actively shedding at the time of sampling, due to immunity provided by the earlier LPAIV infection, especially in free-range birds with increased and relatively close contact compared to caged birds.

We hypothesize that the LPAIV spread from Shed 4 into Shed 5 due to the very high seroconversion of birds in this group, and at the time of cull a significant proportion of birds were actively shedding HPAIV. It is speculated that at the time of cull, all susceptible birds that had not been exposed to LPAIV in Shed 5, had already died due to HPAIV infection. This is based on the assumption that susceptible birds, without prior LPAIV infection, would rapidly succumb to HPAIV infection and that birds that had been infected with the LPAIV may have been shedding virus at the time of sampling [8,25,26]. The peak HI titers in Sheds 5 and 6 (both 4096), which were the highest of all the sheds, suggest that the immune responses of the birds in these sheds were primed by the LPAIV infection, and then boosted when exposed to the HPAIV at a later time.

Birds in Shed 1 were exposed to LPAIV and a large proportion of the birds seroconverted, however LPAIV transmission within the group was incomplete. This was potentially due to the caged system in place in this shed, leaving a significant population (approximately 50%) of naïve birds which would have been susceptible should the virus mutate into a HPAIV form. Shed 1 was also one of only three sheds where the LP CS motif was identified, however, the additional detection of the HP_R_ motif suggests that a LPAIV to HPAIV mutation event may have occurred in Shed 1. Nevertheless, we cannot exclude Shed 5 as the site of the mutation event since the extent of the mortality in this shed was limited, providing uncertainty around early clinical indicators for the presence of HPAIV. Regardless of location, the mutation from LPAIV to HPAIV is believed to have occurred between 29 and 30 June, with an incubation period of four to seven days prior to increasing clinical indices resulting in formal reporting of clinical suspicion.

Our results indicate that after Shed 1, the LPAIV then spread to Sheds 2, 10 and 11 in a similar timeframe based on the serological profiles. Spread of LPAIV in Shed 2 birds was at lower frequency due to caging and less connectivity compared to free-ranging birds and the HPAIV was potentially introduced to this shed during the LPAIV infection. All of these sheds were subsequently exposed to the HPAIV but based on the number of actively shedding birds at cull, it would appear this was later in the infection course than Sheds 1 and 5. Finally, birds in Shed 12A appeared not to have had exposure to the LPAIV based largely on the relative absence of seropositive birds, and therefore constituted the only group on the IP to which the LPAIV had not spread at the time of intervention. Subsequently, HPAIV reached this shed, but the limited number of birds actively shedding at cull, and the absence of any prior immunity, indicate limited viral spread. This is not unexpected as transmission of HPAIVs within caged housing systems has been demonstrated to be slower compared to free-range systems [27] potentially owing to an indirect mode of transmission between adjacent cages via dust and manure belts.

Genetic analyses of the virus indicated that it was closely related to AIVs from wild birds, and since H7 LPAIVs had been identified in wild waterfowl in early 2015 [28], this suggested a relatively recent introduction from wild birds to poultry. However, in the absence of extensive wild bird surveillance, it was not possible to pin-point the incursion timeframe with greater accuracy. The combination of these genetic analyses, the presence of wild waterfowl on the ponds located on the premises, and the lack of relevant national or international tracings and production records substantiated the hypothesis that the source of the infection was contamination via wild birds.

At the time of the outbreak, there were a total of 87 poultry holdings within the 3 km PZ, containing 67,445 birds including chickens, ducks, geese, guinea fowl, pigeons, turkeys, and quail as well as other birds and exotic species. Within the 10 km SZ, there were a total of 105 poultry holdings, containing 372,750 birds including a similar diversity as above. There were also four bird reserves within 30 km of the IP, as well as a large number of captive and wild gamebirds reported in the surrounding area. Within the extended company, there were a large number of personnel and contacts with other businesses. In total, 108 source and 123 spread tracing tasks were generated dating back to 12 June, based on the OIE precautionary incubation period of 21 days [29], with the highest likelihood for HPAIV spread occurring between 29 June and 8 July. In total, 103 premises were identified as potential contact premises via tracings, including premises most closely associated with the IP within the company. Investigations, consisting of clinical inspection, checks of production records and laboratory testing of eleven submissions from nine premises, resulted in entirely negative findings. No consignments of live birds or hatching eggs/day-old chicks were imported onto the premises or other company-owned premises during the risk period. There was also no evidence of contaminated products being brought onto the premises during the risk period. No further cases of H7N7 AIV were identified in domestic poultry in the UK, despite raised awareness following confirmation of the disease. Assessments of onward transmission both within and outside of the company that owned and ran the premises did not suggest that there had been any spread of infection from the premises.

Previous outbreaks of H7N7 LPAIV were detected in poultry holdings in the UK in 2008 [4] and across Europe: Denmark in 2013 [30], Germany in 2009, 2011, and 2013 [31,32,33,34,35], as well as in The Netherlands in 2011, 2012 and 2013 [36,37,38,39,40], none of which were associated with human cases [41]. In 2015, outbreaks of H7N7 LPAIV were reported in poultry in Germany (March 2015 and June 2015), The Netherlands (two outbreaks in March 2015) [8], and the UK (February 2015) [3]. Germany and The Netherlands both have early warning systems for poultry, as does the UK through the “testing to exclude” scheme [42], as well as wild bird and poultry surveillance schemes and this case study provides further evidence that incursions of H7N7 LPAIV are possible. Nonetheless, early detection is vital to prevent mutation into HPAIV strains that will be more likely to occur in dense poultry populations.

Mutation from LPAIV to HPAIV is correlated with the acquisition of multiple basic amino acids (arginine and lysine) at the CS of the HA protein [43]. This enables HPAIVs to replicate in many tissues causing systemic infection in birds [44]. The instances of a LPAIV mutating to a HPAIV from a single introduction are limited, but has occurred previously for H7N7 AIV in: Australia [45], The Netherlands [46], UK [4] and Germany [8]. In Germany, the H7N7 LPAIV to HPAIV mutation occurred within 45 days and between two premises only 400 m apart [8]. However, the study presented here along with a previous UK study [4], found this transition to occur on a single premises within a matter of days. Of note also is that this study identified two different HP CS motifs, HP_G_ and HP_R_, which it is speculated is the result of further evolution of the HPAIV during transmission through the premises. Isolation of LPAIV/HPAIV isolate pairs is a rare phenomenon, and in the instances of this and the previous UK study, it was not possible to isolate the LPAIV from clinical material, unlike the German H7N7 LPAIV [8]. Such virus pairs are invaluable for investigating the factors that drive the transition from LPAIV to HPAIV in nature. Reverse genetic systems are a viable alternative to investigate such transitions in the absence of a complete LPAIV/HPAIV isolate pair; however, these require whole genome sequence data of the missing component to provide meaningful comparisons. Nevertheless, further work is required to elucidate factors other than the HA CS involved in the transition from LPAIV to HPAIV.

Serological evidence and differing degrees of mortality suggest that the H7N7 LPAIV may have afforded some protection against the HPAIV which would normally cause high mortality. In this outbreak, it appears that the LPAIV primed the immune responses and, when subsequently challenged by the HPAIV, resulted in an anamnestic humoral response to H7. Previous work has demonstrated the ability of LPAIVs to outcompete HPAIVs *in ovo* as well as in experimentally-infected chickens [47]. However, the effect and extent that prior LPAIV infection in chickens has on later HPAIV challenge has not been fully investigated. Such work, together with assessments of the selective pressure imposed by switching from a waterfowl to a gallinaceous host, could provide further information regarding the immune and host factors involved in the LPAIV to HPAIV transition, as well as homo- and heterosubtypic cross-protection between AIVs.

In conclusion, detection of an H7N7 HPAIV in commercial layers, led to the identification of a LPAIV progenitor from clinical material through molecular investigations. Serological evidence identified birds that had seroconverted to H7 AIV, further supporting the hypothesis that a H7N7 LPAIV had circulated on the premises prior to mutation to HPAIV. By investigation of such events and the isolation of LPAIV/HPAIV pairs, a further understanding of the factors that cause this LPAIV to HPAIV transition will be gained, which can then inform interventions to control and limit the threat of such poultry incursions. It is noteworthy that numerous introductions of H7N7 LPAIV to gallinaceous poultry, especially in laying birds (and therefore also older birds) has led to rapid mutation to HPAIV compared to H7 viruses with other neuraminidase types. This could indicate increased propensity of H7N7 LPAIVs to mutate to HPAIV and might be taken into consideration by competent veterinary authorities when imposing control measures.

## Figures and Tables

**Table 1:** Samples were collected from the infected premises (IP) in three separate sample sets. OP, oropharyngeal and C, cloacal.

	Set 1	Set 2	Set 3
Date of submission	7 July 2015	9 July 2015	11–13 July 2015
Sample type and number	11 carcasses	20 OP swabs, per shed 20 C swabs, per shed 20 clotted bloods, per shed	60 OP swabs, per shed 60 C swabs, per shed 60 clotted bloods, per shed 2 carcasses, per shed
Origin of samples	Sheds 10 (N = 5) and 12A (N = 6)	Sheds 1, 2, 10, 11, and 12A	Sheds 1, 2, 4, 5, 6, 7, 10, 11, and 12A

**Table 2:** Real-time reverse-transcription (RRT)-PCR and sequencing results from Sample Set 2. Hemagglutinin (HA) cleavage site (CS) motifs for each shed were determined based on sequencing of individual swabs and/or viral isolates. High pathogenicity (HP)_G_; HA CS motif PEIPRHRK**G**RGLF, HP_R_; HA CS motif PEIPRHRK**R**RGLF.

Shed	Type	Percentage of RRT-PCR Positive Samples at Whole Bird Level (%) (N = 20)				Cleavage Site Motif Detected	AIV Molecular Pathotype
		Influenza A	AOAV-1	H5	H7		
1	Caged	50	0	0	50	PEIPRHRK**R**RGLF	HP_R_
2	Caged	70	0	0	75	PEIPRHRK**G**RGLF	HP_G_
10	Free-range	70	0	0	75	PEIPRHRK**G**RGLF	HP_G_
11	Free-range	65	0	0	90	PEIPRHRK**G**RGLF PEIPRHRK**R**RGLF	HP_G_/HP_R_
12A	Caged	50	0	0	65	PEIPRHRK**R**RGLF	HP_R_

Changes in the HA CS motif are shown in bold.

**Table 3:** RRT-PCR and sequencing results from Sample Set 3. HA CS motifs for each shed were determined based on sequencing of individual swabs. HP_G_; HA CS motif PEIPRHRK**G**RGLF, HP_R_; HA CS motif PEIPRHRK**R**RGLF, LP; HA CS motif PEIPKGRGLF.

Shed	Type	Percentage of RRT-PCR Positive Samples at Whole Bird Level by H7 RRT-PCR (%) (N = 60)	Cleavage Site Motif Detected	AIV Molecular Pathotype
1	Caged	47	**PEIPKGRGLF** PEIPRHRK**R**RGLF	LP/HP_R_
2	Caged	83	**PEIPKGRGLF** PEIPRHRK**R**RGLF	LP/HP_R_
4 *	Free-range	0	**PEIPKGRGLF**	LP
5	Free-range	32	PEIPRHRK**G**RGLF PEIPRHRK**R**RGLF	HP_G_/HP_R_
6	Free-range	3	PEIPRHRK**G**RGLF PEIPRHRK**R**RGLF	HP_G_/HP_R_
7	Free-range	2	PEIPRHRK**G**RGLF PEIPRHRK**R**RGLF	HP_G_/HP_R_
10	Free-range	52	PEIPRHRK**G**RGLF PEIPRHRK**R**RGLF	HP_G_/HP_R_
11	Free-range	90	PEIPRHRK**R**RGLF	HP_R_
12A	Caged	38	PEIPRHRK**G**RGLF PEIPRHRK**R**RGLF	HP_G_/HP_R_

* No swabs from Shed 4 resulted in H7 RRT-PCR Ct values < 36.0, but CS sequence was obtained from a swab with a Ct value of 38.17. Changes in the HA CS motif are shown in bold.

**Table 4:** Summary of the HA CS motifs detected across Sample Sets 1, 2, and 3. HA CS motifs for each shed were determined based on sequencing of individual swabs, pooled tissues, or viral isolates. HP_G_; HA CS motif PEIPRHRK**G**RGLF, HP_R_; HA CS motif PEIPRHRK**R**RGLF, LP; HA CS motif PEIPKGRGLF, -; not determined

Shed	Type	Tissue RRT-PCR Results (2 Carcasses per Shed)		
		Set 1	Set 2	Set 3
		7 July 2015	9 July 2015	11–13 July 2015
1	Caged	-	HP_R_	LP/HP_R_
2	Caged	-	HP_G_	LP/HP_R_
4	Free-range	-	-	LP
5	Free-range	-	-	HP_G_/HP_R_
6	Free-range	-	-	HP_G_/HP_R_
7	Free-range	-	-	HP_G_/HP_R_
10	Free-range	HP_G_	HP_G_	HP_G_/HP_R_
11	Free-range	HP_R_	HP_G_/HP_R_	HP_R_
12A	Caged	-	HP_R_	HP_G_/HP_R_

**Table 5:** Hemagglutination inhibition (HI) results from Sample Set 2. The number of sera tested from each shed is provided in brackets.

Shed	Type	Percentage Antibody Positive Samples by HI at Whole Bird Level (%)		
		AOAV-1	H5 ^1^	H7 ^2^
1 (N = 14)	Caged	0	0	43
2 (N = 17)	Caged	0	0	12
10 (N = 18)	Free-range	0	0	33
11 (N = 20)	Free-range	0	0	45
12A (N = 15)	Caged	0	0	7

^1^ Sera were only tested using the primary H5 AIV antigen, H5N3. ^2^ Sera were determined positive to H7 AIV antigens if seropositive towards either the primary (H7N7) and/or secondary (H7N1) AIV antigens.

**Table 6:** HI results from Sample Set 3. The number of sera tested from each shed is provided in brackets.

Shed	Type	Percentage H7N1 Antibody Positive Samples by HI at Whole Bird Level (%)
1 (N = 54)	Caged	54
2 (N = 60)	Caged	8
4 (N = 60)	Free-range	90
5 (N = 60)	Free-range	100
6 (N = 58)	Free-range	91
7 (N = 59)	Free-range	41
10 (N = 60)	Free-range	82
11 (N = 60)	Free-range	60
12A (N = 60)	Caged	2

**Table 7:** Infection and antibody status of birds on the IP. The RRT-PCR and HI results from Sample Sets 2 and 3 were combined to determine the infection and antibody status of the birds and placed them into one of the four categories. Only birds from which RRT-PCR values and HI titers were determined were included.

Shed	Type	Birds within Each Category (%)				AIV Molecular Pathotype
		Category I	Category II	Category III	Category IV	
		H7 RRT-PCR Negative	H7 RRT-PCR Positive	H7 RRT-PCR Positive	H7 RRT-PCR Negative	
		H7 AIV Seronegative	H7 AIV Seronegative	H7 AIV Seropositive	H7 AIV Seropositive	
1	Caged	21.6	31.1	13.5	33.8	LP/HP_R_
2	Caged	15.0	76.3	5.0	3.8	LP/HP_G_/HP_R_
4	Free-range	10.0	0.0	0.0	90.0	LP
5	Free-range	0.0	0.0	31.7	68.3	HP_G_/HP_R_
6	Free-range	8.6	0.0	1.7	89.7	HP_G_/HP_R_
7	Free-range	59.3	0.0	1.7	39.0	HP_G_/HP_R_
10	Free-range	1.3	28.2	28.2	42.3	HP_G_/HP_R_
11	Free-range	1.3	42.5	47.5	8.8	HP_G_/HP_R_
12A	Caged	52.5	45.0	0.0	2.5	HP_G_/HP_R_

## Data Availability

Source data are available upon request.

## Supplementary Materials

The following are available online at https://www.mdpi.com/1999-4915/13/2/259/s1. Table S1: Immunohistochemical distribution of influenza A viral nucleoprotein antigen in carcasses from Sample Sets 1 and 3. Table S2: RRT-PCR and sequencing results from Sample Set 1. Table S3: Summary of the carcass tissues from Sample Set 3 with RRT-PCR results. Table S4: Summary of the type isolate viruses obtained from each shed in Sample Set 2. Table S5: Polymorphisms observed in A/chicken/England/26352/2015 that may confer adaptation to mammalian species or altered susceptibility to existing antivirals. Figure S1: Previous UK H7N7 outbreaks were also shed via the gastrointestinal route. Figure S2: A/chicken/England/26352/2015 is related to contemporary H7N7 or other wild bird AIVs from Europe. Figure S3: Sera from the outbreak demonstrated greater affinity to the H7N1 AIV antigen.
