# Supplementary material for: H7N7 Avian Influenza Virus Mutation from Low to High Pathogenicity on a Layer Chicken Farm in the UK
Source: Viruses. 2021 Feb 8;13(2):259. doi: 10.3390/v13020259 (PMC7914596; doi:10.3390/v13020259)
Supplement: Supplementary file 1 [file viruses-13-00259-s001.pdf]

H7N7 avian influenza virus mutation from low to high pathogenicity on a layer chicken farm in the UK

Supplementary Materials

Table S1. Immunohistochemical distribution of influenza A viral nucleoprotein antigen in carcasses from Sample Sets 1 and 3. Additional observations also included: N, necrosis; E, encephalitis; P, necrotizing pancreatitis and C, coelomitis/peritonitis – chronic active. N/A, not available; -, negative; +/-, minimal or rare antigen labelling; +, small number of labelled cells or antigen; ++, moderate antigen labelling; +++ abundant antigen labelling.

**Sample Set**	**1**		**3**																	
**Shed**	**10**	**12A**	**1**		**2**		**4**		**5**		**6**		**7**		**10**		**11**		**12A**	
**Bird**	**Mix**	**Mix**	**1**	**2**	**1**	**2**	**1**	**2**	**1**	**2**	**1**	**2**	**1**	**2**	**1**	**2**	**1**	**2**	**1**	**2**
Heart	+	N/A	-	-	-	-	-	-	-		-	-	-	-	-	-	-	-	-	-
Skin	-	N/A	-	-	N/A	N/A	-	-	-	-	-	-	-	-	-	N/A	-	N/A	-	-
Feather follicles	N/A	N/A	N/A	N/A	N/A	N/A	N/A	N/A	N/A	N/A	N/A	N/A	N/A	N/A	N/A	N/A	-	N/A	N/A	N/A
Skeletal muscle	-	N/A	-	-	-	-	-	-	-	-	-	-	-	N/A	-	-	-	-	-	-
Spleen	+++<br>N	+++<br>N	-	N/A	+++<br>N	+	-	-	-	-	+	-	-	-	+	-	+<br>N	+<br>N	+<br>N	-
Cecal Tonsil	+++	+	N/A	-	+	-	-	-	-	-	+	-	-	-	N/A	++	-	-	+	-
Brain	-	-	-	-	N/A	-	-	-	-	-	-	-	+/-	-	-	-	-	++<br>E	-	-
Kidney	++	+	-	-	N/A	-	-	-	-	-	-	-	-	-	-	-	-	+<br>N	+	-
Ovary	+	N/A	-	-	++	-	-	-	+	+	+	-	-	-	+	-	N/A	+	+<br>C	-
Oviduct		N/A	N/A	-	N/A	N/A	N/A	N/A	N/A	N/A	N/A	N/A	N/A	-	++	N/A	N/A	++	N/A	N/A
Lung	+	-	-	-	+	-	-	-	-	-	+	-	-	+/-	+	+	-	++	-	-
Trachea / Bronchi	++	+	-	-	N/A	N/A	-	-	-	-	+	-	-		+	-	-	-	N/A	-
Nasal turbinate	-	-	-	-	+++	-	-	-	-	-	-	-	-	-	-	-	-	-	-	-
Air Sacs	+	+	N/A	N/A	N/A	N/A	-	-	+	N/A	+	-	-	+/-	N/A	N/A	N/A	N/A	N/A	-
Pancreas	N/A	N/A	-	-	+	-	-	-	-		-	-	-	-	+	-	-	++<br>P	-	-
Duodenum	+<br>C	N/A	-	-	+	-	-	-	-	-	+	-	-	-	N/A	-	-	-	-	-

Table S2 cont. Immunohistochemical distribution of influenza A viral nucleoprotein antigen in carcasses from Sample Sets 1 and 3. Additional observations also included: N, necrosis; E, encephalitis; P, necrotizing pancreatitis and C, coelomitis/peritonitis – chronic active. N/A, not available; -, negative; +/-, minimal or rare antigen labelling; +, small number of labelled cells or antigen; ++, moderate antigen labelling; +++ abundant antigen labelling.

**Sample Set**	**1**		**3**																					
**Shed**	**10**	**12A**	**1**		**2**		**4**		**5**		**6**		**7**		**10**		**11**		**12A**	
**Bird**	**Mix**	**Mix**	**1**	**2**	**1**	**2**	**1**	**2**	**1**	**2**	**1**	**2**	**1**	**2**	**1**	**2**	**1**	**2**	**1**	**2**
Liver	+	+	-	-	++	-	-	-	-	-	-	-	-	-	-	-	-	N/A	++<br>N	-
Jejunum	++<br>C	+	-	-	+	-	-	-	-	-	-	-	-	-	+<br>C	-	-	+<br>C	+<br>C	-

Table S2. RRT-PCR and sequencing results from Sample Set 1. HA CS motifs for each shed were determined based on sequencing of pooled tissues. HP_G; HA CS motif PEIPRHRK**G**RGLF, HP_R; HA CS motif PEIPRHRK**R**RGLF, +; RRT-PCR positive, -; RRT-PCR negative, nt; not tested.

Shed	Type	Tissue	Tissue RRT-PCR Results				Cleavage Site Motif Detected	AIV Molecular Pathotype
			Influenza A	AOAV-1	H5	H7		
10	Free-range	Trachea (N=2)	+	-	-	+	nt	nt
10	Free-range	Cecal tonsil (N=2)	+	-	-	+	PEIPRHRK**G**RGLF	HP_G
12A	Caged	Trachea (N=4)	+	-	-	+	nt	nt
12A	Caged	Cecal tonsil (N=4)	+	-	-	+	PEIPRHRK**R**RGLF	HP_R
12A	Caged	Spleen (N=4)	+	-	-	+	nt	nt
12A	Caged	Proventriculus (N=1)	+	-	-	+	nt	nt

Changes in the HA CS motif are shown in bold.

Table S3. Summary of the carcass tissues from Sample Set 3 with RRT-PCR results. HA CS motifs for each shed were determined based on sequencing of individual swabs. HP_G; HA CS motif PEIPRHRK**G**RGLF, HP_R; HA CS motif PEIPRHRK**R**RGLF, LP; HA CS motif PEIPKGRGLF, +; RRT-PCR positive, -; RRT-PCR negative.

Shed	Type	AIV Molecular Pathotype	Tissue RRT-PCR Results (2 carcasses per shed)			
			Brain	Lung and Trachea	Viscera	Intestine
1	Caged	LP/HP_R	-	-	-	-
2	Caged	LP/HP_R	+	+	+	+
4	Free-range	LP	-	-	-	-
5	Free-range	HP_G/HP_R	-	-	-	-
6	Free-range	HP_G/HP_R	-	+	+	+
7	Free-range	HP_G/HP_R	-	-	-	-
10	Free-range	HP_G/HP_R	-	+	+	+
11	Free-range	HP_R	+	+	+	+

Table S4. Summary of the type isolate viruses obtained from each shed in Sample Set 2.

Shed	Type	Name of virus isolate
1	Caged	A/chicken/England/26350/2015
2	Caged	A/chicken/England/26352/2015
10	Free-range	A/chicken/England/26346/2015
11	Free-range	A/chicken/England/26348/2015
12A	Caged	A/chicken/England/26354/2015

Table S5. Polymorphisms observed in A/chicken/England/26352/2015 that may confer adaptation to mammalian species or altered susceptibility to existing antivirals.

Protein	Amino Acid Position/Motif[1]	Phenotypic Consequences	Reference
PB2	Collective mutations: Leu89Val, Gly309Asp, Thr339Lys, Arg477Gly, Ile495Val, Lys627Glu, Ala676Thr	Enhanced polymerase activity and increased virulence in mice	[48]
PB1-F2	Asn66Ser	Increased virulence, replication efficiency and antivirus response in mice	[49-51]
HA[2]	Ser128Ala	Increased binding to human receptors	[52]
HA[2]	Thr151Ala	Loss of glycosylation increased binding to human receptors	[52]
HA[2]	Gly177Val	Increased binding to human receptors	[52]
NA[3]	His274Arg	Reduced susceptibility to oseltamivir and peramivir	[53-59]
M1	Asn30Asp	Increased virulence in mice	[60]
M1	Thr215Ala	Increased virulence in mice	[61, 62]
NS1	Ile101Met	Increased virulence in mice	[63, 64]
NS1	222-225 (presence of PDZ ligand domain)	Increased virulence in mice	[65]

Reference for Table S5.

48. Li, J., M. Ishaq, M. Prudence, X. Xi, T. Hu, Q. Liu, and D. Guo, Single mutation at the amino acid position 627 of PB2 that leads to increased virulence of an H5N1 avian influenza virus during adaptation in mice can be compensated by multiple mutations at other sites of PB2. *Virus Res,* 2009. **144**(1-2): p. 123-9. 10.1016/j.virusres.2009.04.008.

49. Schmolke, M., B. Manicassamy, L. Pena, T. Sutton, R. Hai, Z.T. Varga, B.G. Hale, J. Steel, D.R. Perez, and A. Garcia-Sastre, Differential contribution of PB1-F2 to the virulence of highly pathogenic H5N1 influenza A virus in mammalian and avian species. *PLoS Pathog,* 2011. **7**(8): p. e1002186. 10.1371/journal.ppat.1002186.

50. Conenello, G.M., D. Zamarin, L.A. Perrone, T. Tumpey, and P. Palese, A single mutation in the PB1-F2 of H5N1 (HK/97) and 1918 influenza A viruses contributes to increased virulence. *PLoS Pathog,* 2007. **3**(10): p. 1414-21. 10.1371/journal.ppat.0030141.

51. Conenello, G.M., J.R. Tisoncik, E. Rosenzweig, Z.T. Varga, P. Palese, and M.G. Katze, A single N66S mutation in the PB1-F2 protein of influenza A virus increases virulence by inhibiting the early interferon response in vivo. *J Virol,* 2011. **85**(2): p. 652-62. 10.1128/jvi.01987-10.

52. Kageyama, T., S. Fujisaki, E. Takashita, H. Xu, S. Yamada, Y. Uchida, G. Neumann, T. Saito, Y. Kawaoka, and M. Tashiro, Genetic analysis of novel avian A(H7N9) influenza viruses isolated from patients in China, February to April 2013. *Euro Surveill,* 2013. **18**(15): p. 20453.

53. Gubareva, L.V., R.G. Webster, and F.G. Hayden, Comparison of the activities of zanamivir, oseltamivir, and RWJ-270201 against clinical isolates of influenza virus and neuraminidase inhibitor-resistant variants. *Antimicrob Agents Chemother,* 2001. **45**(12): p. 3403-8. 10.1128/aac.45.12.3403-3408.2001.

54. Le, Q.M., M. Kiso, K. Someya, Y.T. Sakai, T.H. Nguyen, K.H. Nguyen, N.D. Pham, H.H. Ngyen, S. Yamada, Y. Muramoto, et al., Avian flu: isolation of drug-resistant H5N1 virus. *Nature,* 2005. **437**(7062): p. 1108. 10.1038/4371108a.

55. de Jong, M.D., T.T. Tran, H.K. Truong, M.H. Vo, G.J. Smith, V.C. Nguyen, V.C. Bach, T.Q. Phan, Q.H. Do, Y. Guan, et al., Oseltamivir resistance during treatment of influenza A (H5N1) infection. *N Engl J Med,* 2005. **353**(25): p. 2667-72. 10.1056/NEJMoa054512.

56. Iatsyshina, S.B., A.M. Shestopalov, V.A. Evseenko, T.S. Astakhova, S.I. Braslavskaia, V.A. Ternovoi, T. Kondrat'eva, A. Alekseev, S.I. Zolotykh, N. Rassadkin Iu, et al., [Isolation and molecular characterization of the influenza virus A/H5N1 strains isolated during outbreak of avian influenza among birds in the European part of Russia in 2005: strain with ozeltamivir-resistance mutation was found]. *Mol Gen Mikrobiol Virusol,* 2008(1): p. 26-34.

57. Hill, A.W., R.P. Guralnick, M.J. Wilson, F. Habib, and D. Janies, Evolution of drug resistance in multiple distinct lineages of H5N1 avian influenza. *Infect Genet Evol,* 2009. **9**(2): p. 169-78. 10.1016/j.meegid.2008.10.006.
58. Hurt, A.C., J.K. Holien, and I.G. Barr, In vitro generation of neuraminidase inhibitor resistance in A(H5N1) influenza viruses. *Antimicrob Agents Chemother,* 2009. **53**(10): p. 4433-40. 10.1128/aac.00334-09.
59. Govorkova, E.A., N.A. Ilyushina, D.A. Boltz, A. Douglas, N. Yilmaz, and R.G. Webster, Efficacy of oseltamivir therapy in ferrets inoculated with different clades of H5N1 influenza virus. *Antimicrob Agents Chemother,* 2007. **51**(4): p. 1414-24. 10.1128/aac.01312-06.
60. Fan, S., G. Deng, J. Song, G. Tian, Y. Suo, Y. Jiang, Y. Guan, Z. Bu, Y. Kawaoka, and H. Chen, Two amino acid residues in the matrix protein M1 contribute to the virulence difference of H5N1 avian influenza viruses in mice. *Virology,* 2009. **384**(1): p. 28-32. 10.1016/j.virol.2008.11.044.
61. Smeenk, C.A., K.E. Wright, B.F. Burns, A.J. Thaker, and E.G. Brown, Mutations in the hemagglutinin and matrix genes of a virulent influenza virus variant, A/FM/1/47-MA, control different stages in pathogenesis. *Virus Res,* 1996. **44**(2): p. 79-95. 10.1016/0168-1702(96)01329-9.
62. Brown, E.G. and J.E. Bailly, Genetic analysis of mouse-adapted influenza A virus identifies roles for the NA, PB1, and PB2 genes in virulence. *Virus Res,* 1999. **61**(1): p. 63-76. 10.1016/s0168-1702(99)00027-1.
63. Kuo, R.L. and R.M. Krug, Influenza a virus polymerase is an integral component of the CPSF30-NS1A protein complex in infected cells. *J Virol,* 2009. **83**(4): p. 1611-6. 10.1128/jvi.01491-08.
64. Spesock, A., M. Malur, M.J. Hossain, L.M. Chen, B.L. Njaa, C.T. Davis, A.S. Lipatov, I.A. York, R.M. Krug, and R.O. Donis, The virulence of 1997 H5N1 influenza viruses in the mouse model is increased by correcting a defect in their NS1 proteins. *J Virol,* 2011. **85**(14): p. 7048-58. 10.1128/jvi.00417-11.
65. Jackson, D., M.J. Hossain, D. Hickman, D.R. Perez, and R.A. Lamb, A new influenza virus virulence determinant: the NS1 protein four C-terminal residues modulate pathogenicity. *Proc Natl Acad Sci U S A,* 2008. **105**(11): p. 4381-6. 10.1073/pnas.0800482105.

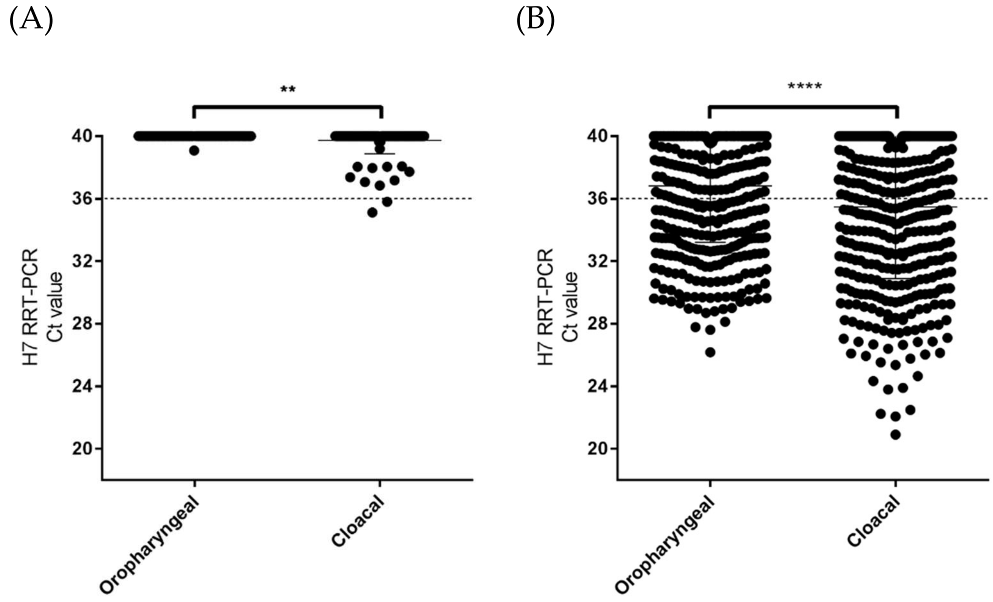

Figure S1. Previous UK H7N7 outbreaks were also shed via the gastrointestinal route. OP and cloacal swabs collected during **(A)** the H7N7 LPAIV outbreak in February 2015 [3] (N=120, per swab type) and **(B)** a H7N7 HPAIV outbreak in 2008 [4] (N=441, per swab type) were tested by H7 RRT-PCR and the level of H7 RNA compared using a Paired T-test. ** p<0.01 and **** p<0.0001.

(A)

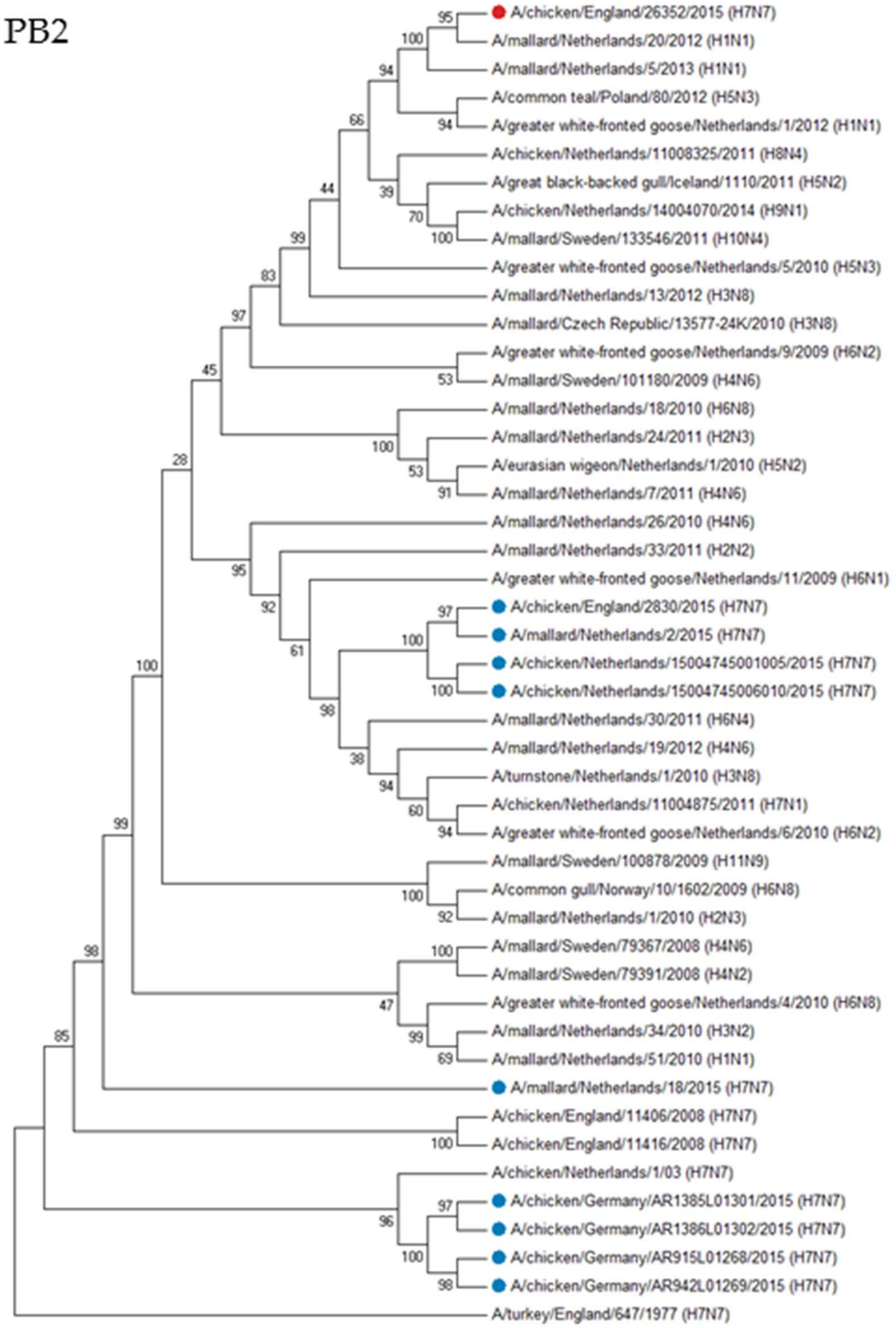

PB2
A/chicken/England/26352/2015 (H7N7)
A/mallard/Netherlands/20/2012 (H1N1)
A/mallard/Netherlands/5/2013 (H1N1)
A/common teal/Poland/80/2012 (H5N3)
A/greater white-fronted goose/Netherlands/1/2012 (H1N1)
A/chicken/Netherlands/11008325/2011 (H8N4)
A/great black-backed gull/Iceland/1110/2011 (H5N2)
A/chicken/Netherlands/14004070/2014 (H9N1)
A/mallard/Sweden/133546/2011 (H10N4)
A/greater white-fronted goose/Netherlands/5/2010 (H5N3)
A/mallard/Netherlands/13/2012 (H3N8)
A/mallard/Czech Republic/13577-24K/2010 (H3N8)
A/greater white-fronted goose/Netherlands/9/2009 (H6N2)
A/mallard/Sweden/101180/2009 (H4N6)
A/mallard/Netherlands/18/2010 (H6N8)
A/mallard/Netherlands/24/2011 (H2N3)
A/eurasian wigeon/Netherlands/1/2010 (H5N2)
A/mallard/Netherlands/7/2011 (H4N6)
A/mallard/Netherlands/26/2010 (H4N6)
A/mallard/Netherlands/33/2011 (H2N2)
A/greater white-fronted goose/Netherlands/11/2009 (H6N1)
A/chicken/England/2830/2015 (H7N7)
A/mallard/Netherlands/2/2015 (H7N7)
A/chicken/Netherlands/15004745001005/2015 (H7N7)
A/chicken/Netherlands/15004745006010/2015 (H7N7)
A/mallard/Netherlands/30/2011 (H6N4)
A/mallard/Netherlands/19/2012 (H4N6)
A/turnstone/Netherlands/1/2010 (H3N8)
A/chicken/Netherlands/11004875/2011 (H7N1)
A/greater white-fronted goose/Netherlands/6/2010 (H6N2)
A/mallard/Sweden/100878/2009 (H11N9)
A/common gull/Norway/10/1602/2009 (H6N8)
A/mallard/Netherlands/1/2010 (H2N3)
A/mallard/Sweden/79367/2008 (H4N6)
A/mallard/Sweden/79391/2008 (H4N2)
A/greater white-fronted goose/Netherlands/4/2010 (H6N8)
A/mallard/Netherlands/34/2010 (H3N2)
A/mallard/Netherlands/51/2010 (H1N1)
A/mallard/Netherlands/18/2015 (H7N7)
A/chicken/England/11406/2008 (H7N7)
A/chicken/England/11416/2008 (H7N7)
A/chicken/Netherlands/1/03 (H7N7)
A/chicken/Germany/AR1385L01301/2015 (H7N7)
A/chicken/Germany/AR1386L01302/2015 (H7N7)
A/chicken/Germany/AR915L01268/2015 (H7N7)
A/chicken/Germany/AR942L01269/2015 (H7N7)
A/turkey/England/647/1977 (H7N7)
95
100
94
94
66
39
70
100
44
99
83
97
53
45
100
53
91
28
95
92
97
100
61
100
98
38
94
60
94
100
100
92
99
100
98
47
99
69
85
100
97
96
100
98

(B)

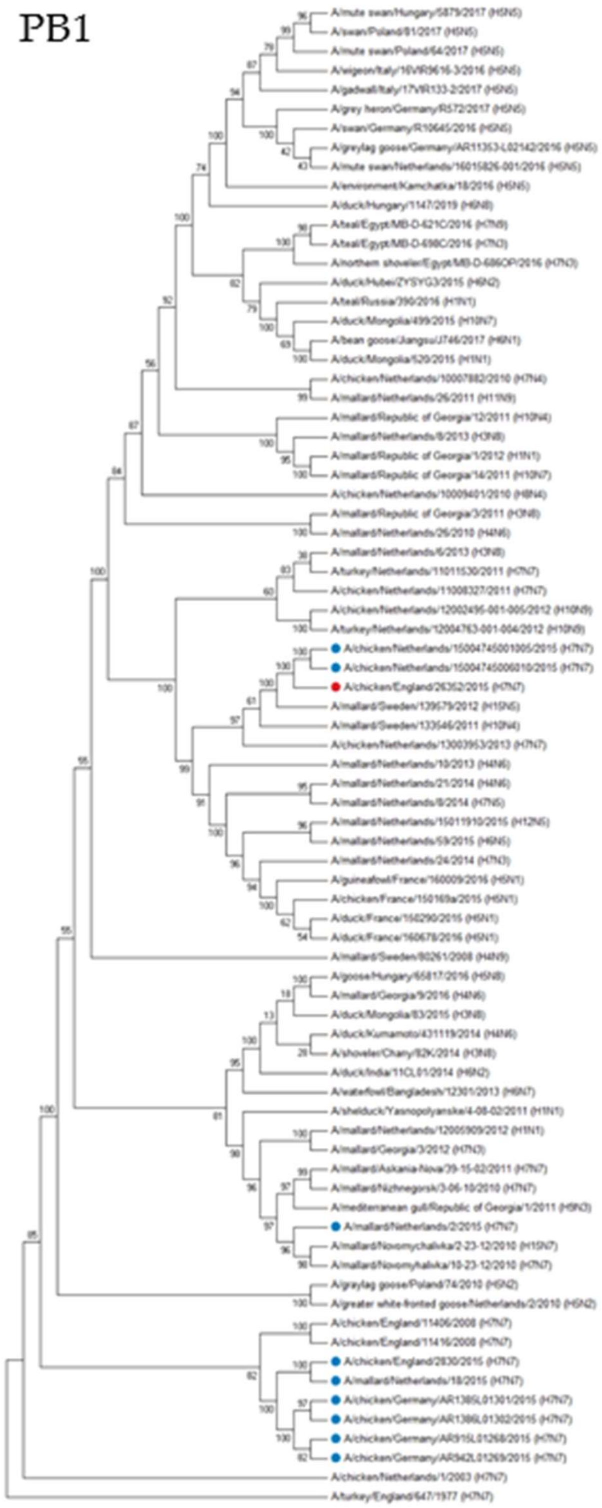
PB1
A/mute swan/Hungary/5879/2017 (H5N5)
A/swan/Poland/81/2017 (H5N5)
A/mute swan/Poland/64/2017 (H5N5)
A/wigeon/Italy/16VIR9616-3/2016 (H5N5)
A/gadwall/Italy/17VIR133-2/2017 (H5N5)
A/grey heron/Germany/R572/2017 (H5N5)
A/swan/Germany/R10645/2016 (H5N5)
A/greylag goose/Germany/AR11353-L02142/2016 (H5N5)
A/mute swan/Netherlands/16015826-001/2016 (H5N5)
A/environment/Kamchatka/18/2016 (H5N5)
A/duck/Hungary/1147/2019 (H6N8)
A/teal/Egypt/MB-D-621C/2016 (H7N9)
A/teal/Egypt/MB-D-698C/2016 (H7N3)
A/northern shoveler/Egypt/MB-D-686OP/2016 (H7N3)
A/duck/Hubei/ZYSYG3/2015 (H6N2)
A/teal/Russia/390/2016 (H1N1)
A/duck/Mongolia/499/2015 (H10N7)
A/bean goose/Jiangsu/J746/2017 (H6N1)
A/duck/Mongolia/520/2015 (H1N1)
A/chicken/Netherlands/10007882/2010 (H7N4)
A/mallard/Netherlands/26/2011 (H11N9)
A/mallard/Republic of Georgia/12/2011 (H10N4)
A/mallard/Netherlands/8/2013 (H3N8)
A/mallard/Republic of Georgia/1/2012 (H1N1)
A/mallard/Republic of Georgia/14/2011 (H10N7)
A/chicken/Netherlands/10009401/2010 (H8N4)
A/mallard/Republic of Georgia/3/2011 (H3N8)
A/mallard/Netherlands/26/2010 (H4N6)
A/mallard/Netherlands/6/2013 (H3N8)
A/turkey/Netherlands/11011530/2011 (H7N7)
A/chicken/Netherlands/11008327/2011 (H7N7)
A/chicken/Netherlands/12002495-001-005/2012 (H10N9)
A/turkey/Netherlands/12004763-001-004/2012 (H10N9)
A/chicken/Netherlands/15004745001005/2015 (H7N7)
A/chicken/Netherlands/15004745006010/2015 (H7N7)
A/chicken/England/26352/2015 (H7N7)
A/mallard/Sweden/139579/2012 (H15N5)
A/mallard/Sweden/133546/2011 (H10N4)
A/chicken/Netherlands/13003953/2013 (H7N7)
A/mallard/Netherlands/10/2013 (H4N6)
A/mallard/Netherlands/21/2014 (H4N6)
A/mallard/Netherlands/8/2014 (H7N5)
A/mallard/Netherlands/15011910/2015 (H12N5)
A/mallard/Netherlands/59/2015 (H6N5)
A/mallard/Netherlands/24/2014 (H7N3)
A/guineafowl/France/160009/2016 (H5N1)
A/chicken/France/150169a/2015 (H5N1)
A/duck/France/150290/2015 (H5N1)
A/duck/France/160678/2016 (H5N1)
A/mallard/Sweden/80261/2008 (H4N9)
A/goose/Hungary/65817/2016 (H5N8)
A/mallard/Georgia/9/2016 (H4N6)
A/duck/Mongolia/83/2015 (H3N8)
A/duck/Kumamoto/431119/2014 (H4N6)
A/shoveler/Chany/82K/2014 (H3N8)
A/duck/India/11CL01/2014 (H6N2)
A/waterfowl/Bangladesh/12301/2013 (H6N7)
A/shelduck/Yasnopolyanske/4-08-02/2011 (H1N1)
A/mallard/Netherlands/12005909/2012 (H1N1)
A/mallard/Georgia/3/2012 (H7N3)
A/mallard/Askania-Nova/39-15-02/2011 (H7N7)
A/mallard/Nizhnegorsk/3-06-10/2010 (H7N7)
A/mediterranean gull/Republic of Georgia/1/2011 (H9N3)
A/mallard/Netherlands/2/2015 (H7N7)
A/mallard/Novomychalivka/2-23-12/2010 (H15N7)
A/mallard/Novomyhalivka/10-23-12/2010 (H7N7)
A/graylag goose/Poland/74/2010 (H5N2)
A/greater white-fronted goose/Netherlands/2/2010 (H5N2)
A/chicken/England/11406/2008 (H7N7)
A/chicken/England/11416/2008 (H7N7)
A/chicken/England/2830/2015 (H7N7)
A/mallard/Netherlands/18/2015 (H7N7)
A/chicken/Germany/AR1385L01301/2015 (H7N7)
A/chicken/Germany/AR1386L01302/2015 (H7N7)
A/chicken/Germany/AR915L01268/2015 (H7N7)
A/chicken/Germany/AR942L01269/2015 (H7N7)
A/chicken/Netherlands/1/2003 (H7N7)
A/turkey/England/647/1977 (H7N7)

(C)

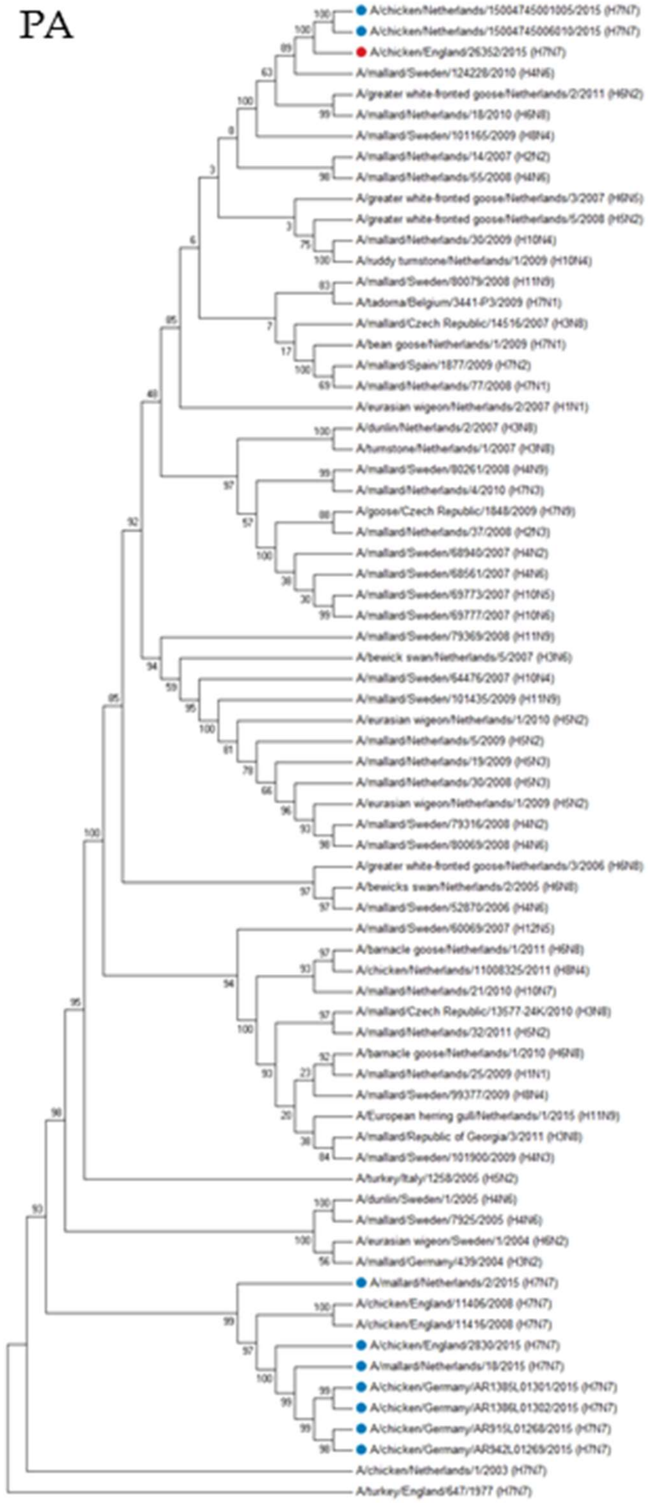

PA
A/chicken/Netherlands/15004745001005/2015 (H7N7)
A/chicken/Netherlands/15004745006010/2015 (H7N7)
A/chicken/England/26352/2015 (H7N7)
A/mallard/Sweden/124228/2010 (H4N6)
A/greater white-fronted goose/Netherlands/2/2011 (H6N2)
A/mallard/Netherlands/18/2010 (H6N8)
A/mallard/Sweden/101165/2009 (H8N4)
A/mallard/Netherlands/14/2007 (H2N2)
A/mallard/Netherlands/55/2008 (H4N6)
A/greater white-fronted goose/Netherlands/3/2007 (H6N5)
A/greater white-fronted goose/Netherlands/5/2008 (H5N2)
A/mallard/Netherlands/30/2009 (H10N4)
A/ruddy turnstone/Netherlands/1/2009 (H10N4)
A/mallard/Sweden/80079/2008 (H11N9)
A/tadoma/Belgium/3441-P3/2009 (H7N1)
A/mallard/Czech Republic/14516/2007 (H3N8)
A/bean goose/Netherlands/1/2009 (H7N1)
A/mallard/Spain/1877/2009 (H7N2)
A/mallard/Netherlands/77/2008 (H7N1)
A/eurasian wigeon/Netherlands/2/2007 (H1N1)
A/dunlin/Netherlands/2/2007 (H3N8)
A/turnstone/Netherlands/1/2007 (H3N8)
A/mallard/Sweden/80261/2008 (H4N9)
A/mallard/Netherlands/4/2010 (H7N3)
A/goose/Czech Republic/1848/2009 (H7N9)
A/mallard/Netherlands/37/2008 (H2N3)
A/mallard/Sweden/68940/2007 (H4N2)
A/mallard/Sweden/68561/2007 (H4N6)
A/mallard/Sweden/69773/2007 (H10N5)
A/mallard/Sweden/69777/2007 (H10N6)
A/mallard/Sweden/79369/2008 (H11N9)
A/bewick swan/Netherlands/5/2007 (H3N6)
A/mallard/Sweden/64476/2007 (H10N4)
A/mallard/Sweden/101435/2009 (H11N9)
A/eurasian wigeon/Netherlands/1/2010 (H5N2)
A/mallard/Netherlands/5/2009 (H5N2)
A/mallard/Netherlands/19/2009 (H5N3)
A/mallard/Netherlands/30/2008 (H5N3)
A/eurasian wigeon/Netherlands/1/2009 (H5N2)
A/mallard/Sweden/79316/2008 (H4N2)
A/mallard/Sweden/80069/2008 (H4N6)
A/greater white-fronted goose/Netherlands/3/2006 (H6N8)
A/bewicks swan/Netherlands/2/2005 (H6N8)
A/mallard/Sweden/52870/2006 (H4N6)
A/mallard/Sweden/60069/2007 (H12N5)
A/barnacle goose/Netherlands/1/2011 (H6N8)
A/chicken/Netherlands/11008325/2011 (H8N4)
A/mallard/Netherlands/21/2010 (H10N7)
A/mallard/Czech Republic/13577-24K/2010 (H3N8)
A/mallard/Netherlands/32/2011 (H5N2)
A/barnacle goose/Netherlands/1/2010 (H6N8)
A/mallard/Netherlands/25/2009 (H1N1)
A/mallard/Sweden/99377/2009 (H8N4)
A/European herring gull/Netherlands/1/2015 (H11N9)
A/mallard/Republic of Georgia/3/2011 (H3N8)
A/mallard/Sweden/101900/2009 (H4N3)
A/turkey/Italy/1258/2005 (H5N2)
A/dunlin/Sweden/1/2005 (H4N6)
A/mallard/Sweden/7925/2005 (H4N6)
A/eurasian wigeon/Sweden/1/2004 (H6N2)
A/mallard/Germany/439/2004 (H3N2)
A/mallard/Netherlands/2/2015 (H7N7)
A/chicken/England/11406/2008 (H7N7)
A/chicken/England/11416/2008 (H7N7)
A/chicken/England/2830/2015 (H7N7)
A/mallard/Netherlands/18/2015 (H7N7)
A/chicken/Germany/AR1385L01301/2015 (H7N7)
A/chicken/Germany/AR1386L01302/2015 (H7N7)
A/chicken/Germany/AR915L01268/2015 (H7N7)
A/chicken/Germany/AR942L01269/2015 (H7N7)
A/chicken/Netherlands/1/2003 (H7N7)
A/turkey/England/647/1977 (H7N7)

(D)

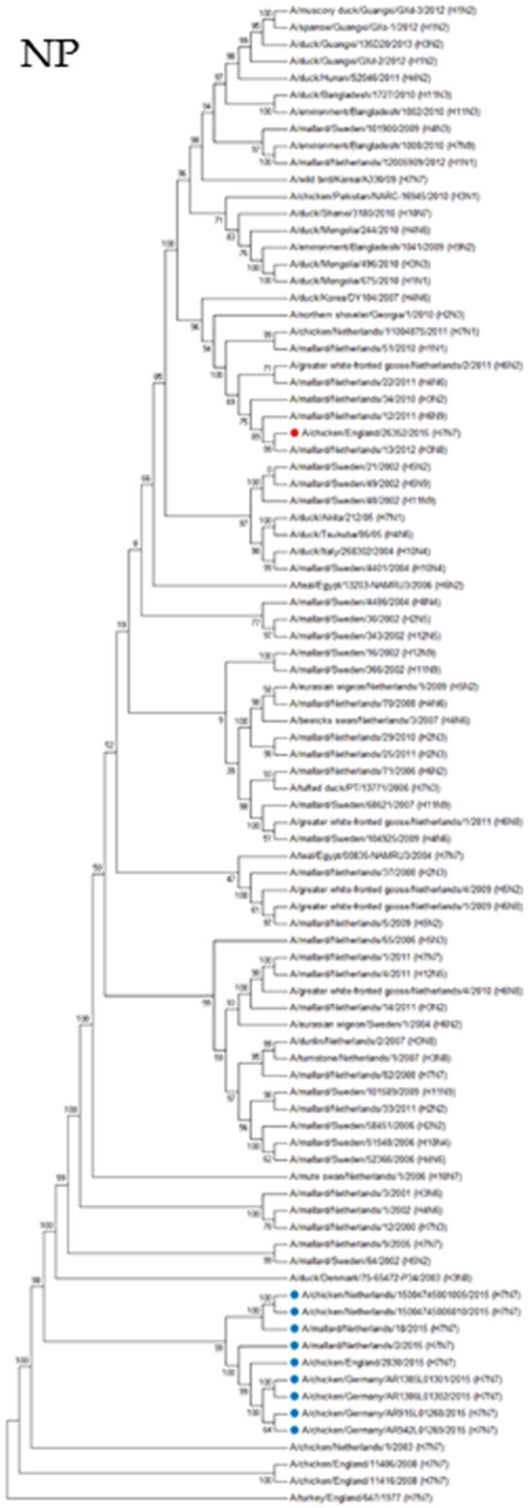

(E)

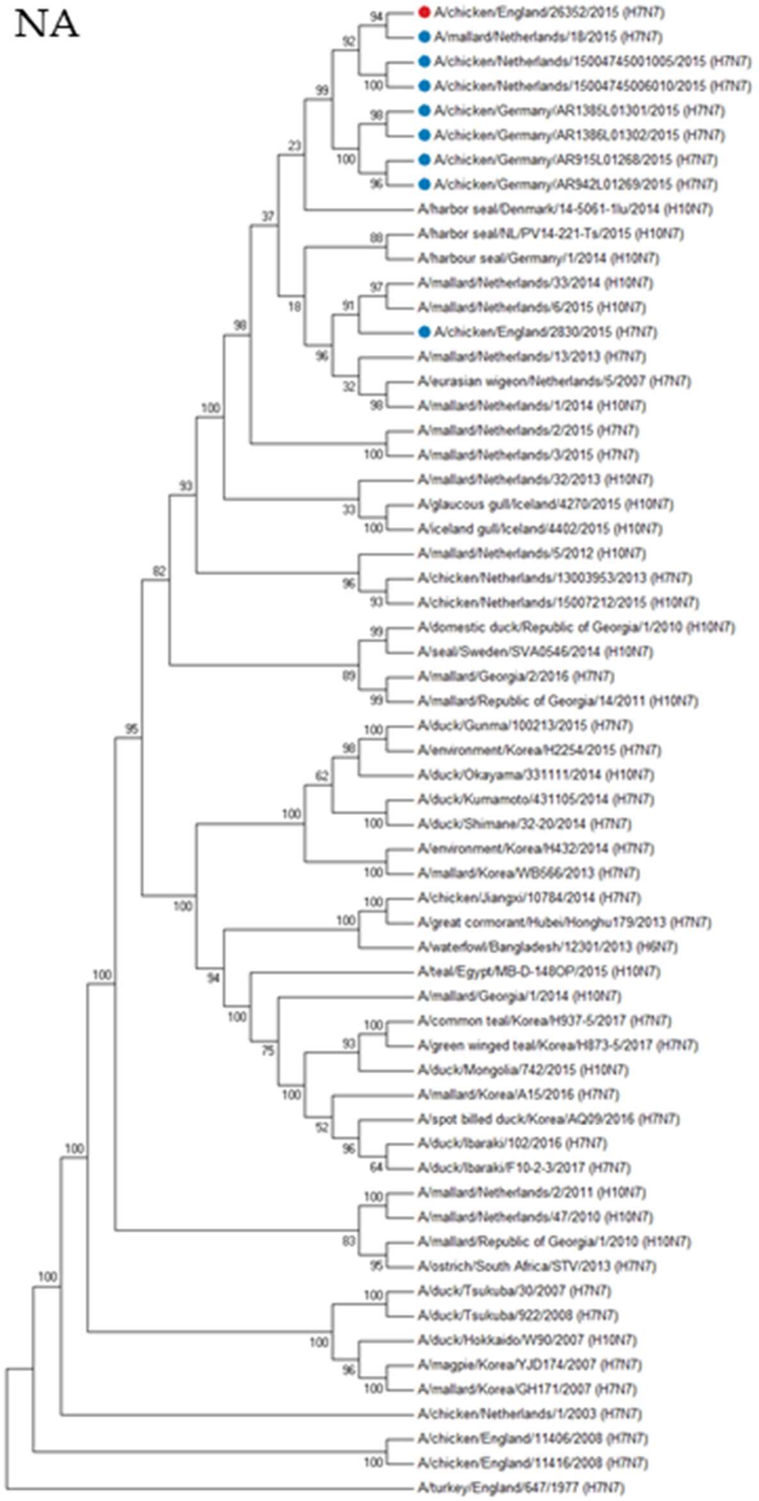
NA
A/chicken/England/26352/2015 (H7N7)
A/mallard/Netherlands/18/2015 (H7N7)
A/chicken/Netherlands/15004745001005/2015 (H7N7)
A/chicken/Netherlands/15004745006010/2015 (H7N7)
A/chicken/Germany/AR1385L01301/2015 (H7N7)
A/chicken/Germany/AR1386L01302/2015 (H7N7)
A/chicken/Germany/AR915L01268/2015 (H7N7)
A/chicken/Germany/AR942L01269/2015 (H7N7)
A/harbor seal/Denmark/14-5061-1lu/2014 (H10N7)
A/harbor seal/NL/PV14-221-Ts/2015 (H10N7)
A/harbour seal/Germany/1/2014 (H10N7)
A/mallard/Netherlands/33/2014 (H10N7)
A/mallard/Netherlands/6/2015 (H10N7)
A/chicken/England/2830/2015 (H7N7)
A/mallard/Netherlands/13/2013 (H7N7)
A/eurasian wigeon/Netherlands/5/2007 (H7N7)
A/mallard/Netherlands/1/2014 (H10N7)
A/mallard/Netherlands/2/2015 (H7N7)
A/mallard/Netherlands/3/2015 (H7N7)
A/mallard/Netherlands/32/2013 (H10N7)
A/glaucous gull/Iceland/4270/2015 (H10N7)
A/iceland gull/Iceland/4402/2015 (H10N7)
A/mallard/Netherlands/5/2012 (H10N7)
A/chicken/Netherlands/13003953/2013 (H7N7)
A/chicken/Netherlands/15007212/2015 (H10N7)
A/domestic duck/Republic of Georgia/1/2010 (H10N7)
A/seal/Sweden/SVA0546/2014 (H10N7)
A/mallard/Georgia/2/2016 (H7N7)
A/mallard/Republic of Georgia/14/2011 (H10N7)
A/duck/Gunma/100213/2015 (H7N7)
A/environment/Korea/H2254/2015 (H7N7)
A/duck/Okayama/331111/2014 (H10N7)
A/duck/Kumamoto/431105/2014 (H7N7)
A/duck/Shimane/32-20/2014 (H7N7)
A/environment/Korea/H432/2014 (H7N7)
A/mallard/Korea/WB566/2013 (H7N7)
A/chicken/Jiangxi/10784/2014 (H7N7)
A/great cormorant/Hubei/Honghu179/2013 (H7N7)
A/waterfowl/Bangladesh/12301/2013 (H6N7)
A/teal/Egypt/MB-D-148OP/2015 (H10N7)
A/mallard/Georgia/1/2014 (H10N7)
A/common teal/Korea/H937-5/2017 (H7N7)
A/green winged teal/Korea/H873-5/2017 (H7N7)
A/duck/Mongolia/742/2015 (H10N7)
A/mallard/Korea/A15/2016 (H7N7)
A/spot billed duck/Korea/AQ09/2016 (H7N7)
A/duck/Ibaraki/102/2016 (H7N7)
A/duck/Ibaraki/F10-2-3/2017 (H7N7)
A/mallard/Netherlands/2/2011 (H10N7)
A/mallard/Netherlands/47/2010 (H10N7)
A/mallard/Republic of Georgia/1/2010 (H10N7)
A/ostrich/South Africa/STV/2013 (H7N7)
A/duck/Tsukuba/30/2007 (H7N7)
A/duck/Tsukuba/922/2008 (H7N7)
A/duck/Hokkaido/W90/2007 (H10N7)
A/magpie/Korea/YJD174/2007 (H7N7)
A/mallard/Korea/GH171/2007 (H7N7)
A/chicken/Netherlands/1/2003 (H7N7)
A/chicken/England/11406/2008 (H7N7)
A/chicken/England/11416/2008 (H7N7)
A/turkey/England/647/1977 (H7N7)

(F)

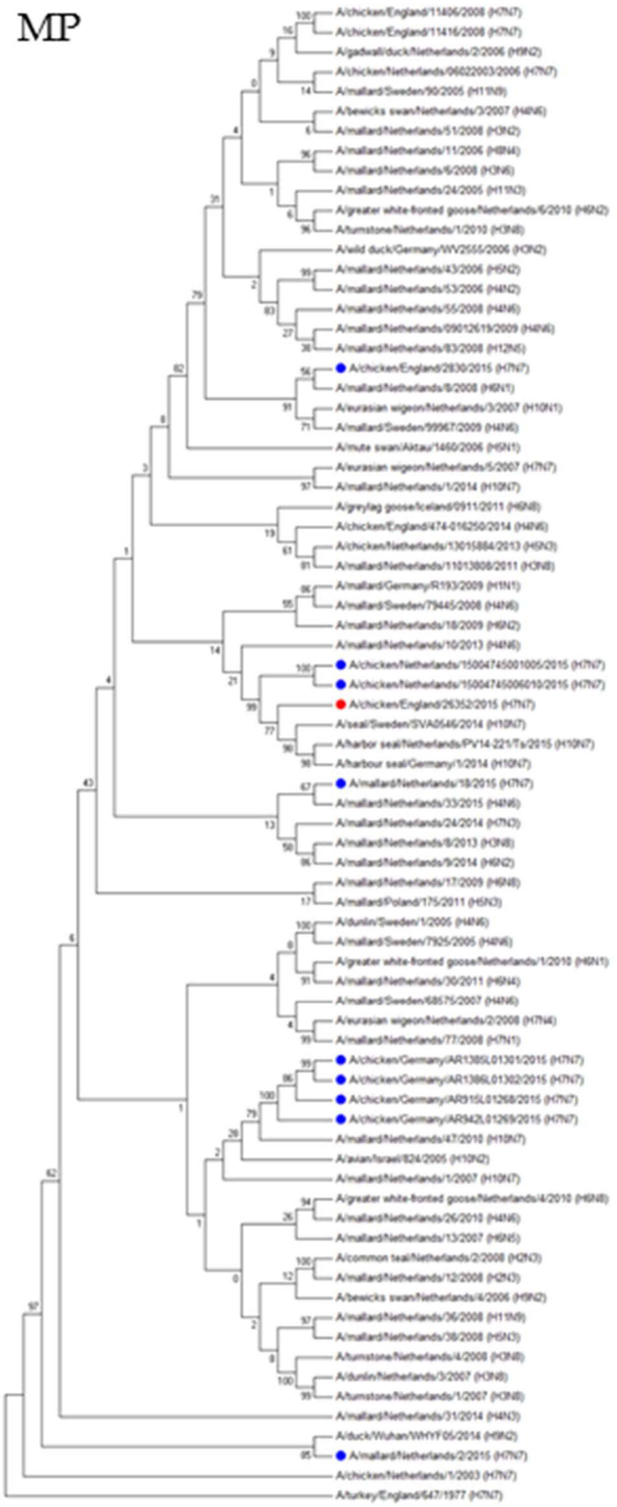
MP
A/chicken/England/11406/2008 (H7N7)
A/chicken/England/11416/2008 (H7N7)
A/gadwall/duck/Netherlands/2/2006 (H9N2)
A/chicken/Netherlands/06022003/2006 (H7N7)
A/mallard/Sweden/90/2005 (H11N9)
A/bewicks swan/Netherlands/3/2007 (H4N6)
A/mallard/Netherlands/51/2008 (H3N2)
A/mallard/Netherlands/11/2006 (H8N4)
A/mallard/Netherlands/6/2008 (H3N6)
A/mallard/Netherlands/24/2005 (H11N3)
A/greater white-fronted goose/Netherlands/6/2010 (H6N2)
A/turnstone/Netherlands/1/2010 (H3N8)
A/wild duck/Germany/WV2555/2006 (H3N2)
A/mallard/Netherlands/43/2006 (H5N2)
A/mallard/Netherlands/53/2006 (H4N2)
A/mallard/Netherlands/55/2008 (H4N6)
A/mallard/Netherlands/09012619/2009 (H4N6)
A/mallard/Netherlands/83/2008 (H12N5)
A/chicken/England/2830/2015 (H7N7)
A/mallard/Netherlands/8/2008 (H6N1)
A/eurasian wigeon/Netherlands/3/2007 (H10N1)
A/mallard/Sweden/99967/2009 (H4N6)
A/mute swan/Aktau/1460/2006 (H5N1)
A/eurasian wigeon/Netherlands/5/2007 (H7N7)
A/mallard/Netherlands/1/2014 (H10N7)
A/greylag goose/Iceland/0911/2011 (H6N8)
A/chicken/England/474-016250/2014 (H4N6)
A/chicken/Netherlands/13015884/2013 (H5N3)
A/mallard/Netherlands/11013808/2011 (H3N8)
A/mallard/Germany/R193/2009 (H1N1)
A/mallard/Sweden/79445/2008 (H4N6)
A/mallard/Netherlands/18/2009 (H6N2)
A/mallard/Netherlands/10/2013 (H4N6)
A/chicken/Netherlands/15004745001005/2015 (H7N7)
A/chicken/Netherlands/15004745006010/2015 (H7N7)
A/chicken/England/26352/2015 (H7N7)
A/seal/Sweden/SVA0546/2014 (H10N7)
A/harbor seal/Netherlands/PV14-221/Ts/2015 (H10N7)
A/harbour seal/Germany/1/2014 (H10N7)
A/mallard/Netherlands/18/2015 (H7N7)
A/mallard/Netherlands/33/2015 (H4N6)
A/mallard/Netherlands/24/2014 (H7N3)
A/mallard/Netherlands/8/2013 (H3N8)
A/mallard/Netherlands/9/2014 (H6N2)
A/mallard/Netherlands/17/2009 (H6N8)
A/mallard/Poland/175/2011 (H5N3)
A/dunlin/Sweden/1/2005 (H4N6)
A/mallard/Sweden/7925/2005 (H4N6)
A/greater white-fronted goose/Netherlands/1/2010 (H6N1)
A/mallard/Netherlands/30/2011 (H6N4)
A/mallard/Sweden/68575/2007 (H4N6)
A/eurasian wigeon/Netherlands/2/2008 (H7N4)
A/mallard/Netherlands/77/2008 (H7N1)
A/chicken/Germany/AR1385L01301/2015 (H7N7)
A/chicken/Germany/AR1386L01302/2015 (H7N7)
A/chicken/Germany/AR915L01268/2015 (H7N7)
A/chicken/Germany/AR942L01269/2015 (H7N7)
A/mallard/Netherlands/47/2010 (H10N7)
A/avian/Israel/824/2005 (H10N2)
A/mallard/Netherlands/1/2007 (H10N7)
A/greater white-fronted goose/Netherlands/4/2010 (H6N8)
A/mallard/Netherlands/26/2010 (H4N6)
A/mallard/Netherlands/13/2007 (H6N5)
A/common teal/Netherlands/2/2008 (H2N3)
A/mallard/Netherlands/12/2008 (H2N3)
A/bewicks swan/Netherlands/4/2006 (H9N2)
A/mallard/Netherlands/36/2008 (H11N9)
A/mallard/Netherlands/38/2008 (H5N3)
A/turnstone/Netherlands/4/2008 (H3N8)
A/dunlin/Netherlands/3/2007 (H3N8)
A/turnstone/Netherlands/1/2007 (H3N8)
A/mallard/Netherlands/31/2014 (H4N3)
A/duck/Wuhan/WHYF05/2014 (H9N2)
A/mallard/Netherlands/2/2015 (H7N7)
A/chicken/Netherlands/1/2003 (H7N7)
A/turkey/England/647/1977 (H7N7)

(G)

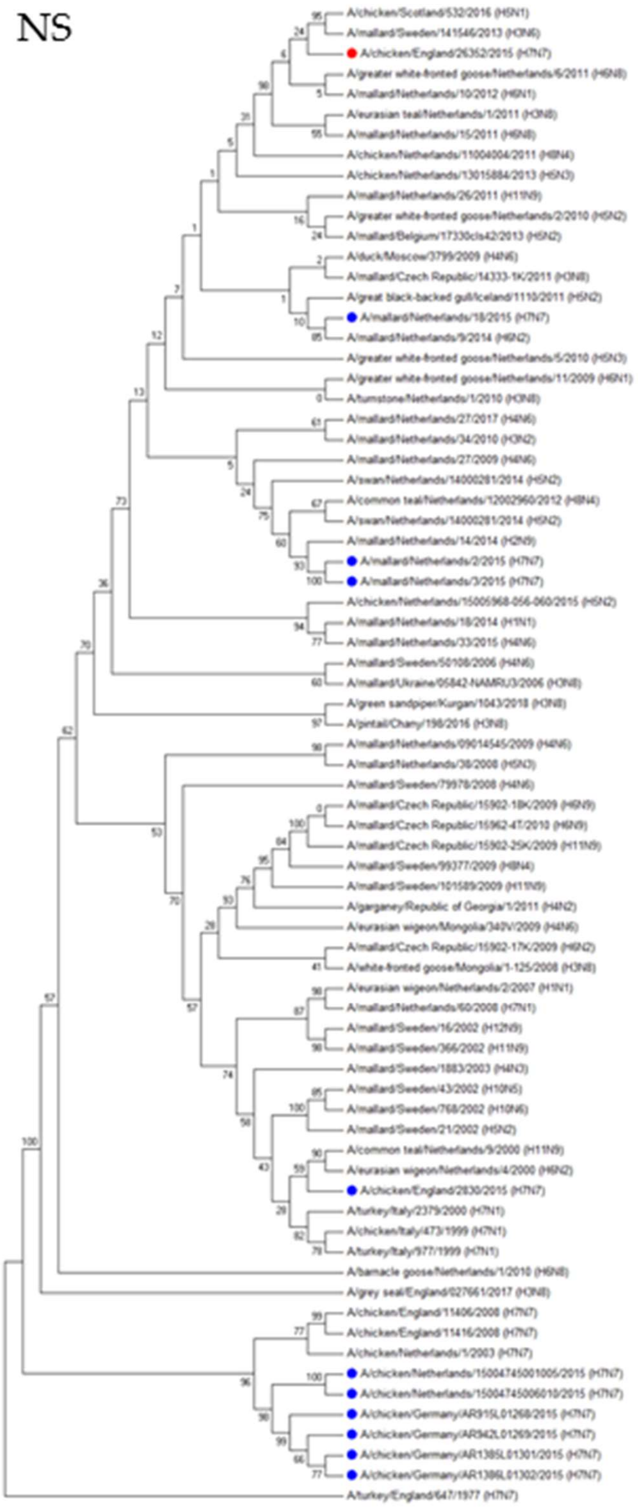

Figure S2. A/chicken/England/26352/2015 is related to AIVs from wild birds. Phylogenetic analysis was conducted using a maximum-likelihood approach for the following influenza A genes: (**A**) PB2, based on the TIM+F+G4 model; (**B**) PB1, based on the TIM+F+G4 model; (**C**) PA, based on the TVM+F+G4 model; (**D**) NP, based on the TVM+F+I+G4 model; (**E**) NA, based on the K3PU+F+G4 model; (**F**) M, based on the K3P+I model and (**G**) NS, based on the HKY+F+I model. A/chicken/England/26352/2015 (H7N7) is indicated by a red circle, whilst contemporary European H7N7 influenza viruses from 2015 are indicated by blue circles. Suitable models were determined using IQ-Tree [17] with ModelFinder [18].

(A)

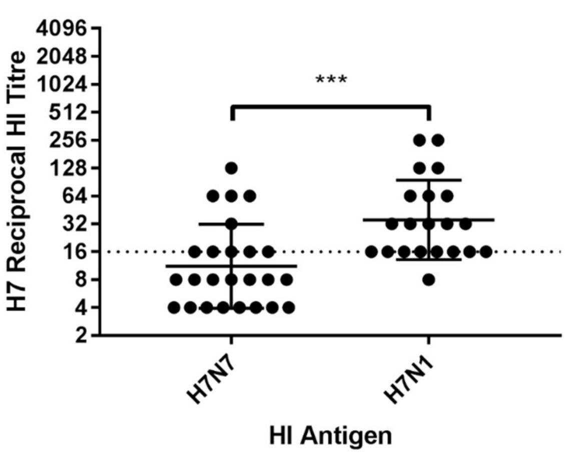

(B)

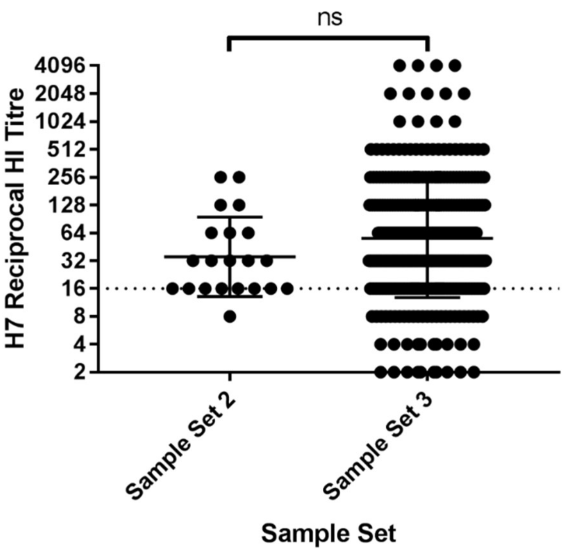

Figure S3. Sera from the outbreak demonstrated greater affinity to the H7N1 AIV antigen. (**A**) Sera from Sample Set 2 were tested by HI against the H7N7 (N=84) and H7N1 (N=21) AIV antigens. The reciprocal HI titers towards these antigens were compared using a Mann-Whitney test. (**B**) The reciprocal HI titers towards the H7N1 AIV antigen of sera from Sample Sets 2 (N=21) and 3 (N=531) were compared using a Mann-Whitney test. ns, not significant $p>0.05$, *** $p<0.001$, ****$p<0.0001$. Graphs show geometric mean ± geometric SD.
